# The dimensionality reductions of environmental variables have a significant effect on the performance of species distribution models

**DOI:** 10.1002/ece3.10747

**Published:** 2023-11-20

**Authors:** Hao‐Tian Zhang, Wen‐Yong Guo, Wen‐Ting Wang

**Affiliations:** ^1^ School of Mathematics and Computer Science Northwest Minzu University Lanzhou China; ^2^ Research Center for Global Change and Complex Ecosystems, School of Ecological and Environmental Sciences East China Normal University Shanghai China; ^3^ Zhejiang Tiantong Forest Ecosystem National Observation and Research Station, School of Ecological and Environmental Sciences East China Normal University Shanghai China

**Keywords:** dimensionality reduction techniques, environmental variables, model complexity, predictive performance, sample sizes, species distribution models

## Abstract

How to effectively obtain species‐related low‐dimensional data from massive environmental variables has become an urgent problem for species distribution models (SDMs). In this study, we will explore whether dimensionality reduction on environmental variables can improve the predictive performance of SDMs. We first used two linear (i.e., principal component analysis (PCA) and independent components analysis) and two nonlinear (i.e., kernel principal component analysis (KPCA) and uniform manifold approximation and projection) dimensionality reduction techniques (DRTs) to reduce the dimensionality of high‐dimensional environmental data. Then, we established five SDMs based on the environmental variables of dimensionality reduction for 23 real plant species and nine virtual species, and compared the predictive performance of those with the SDMs based on the selected environmental variables through Pearson's correlation coefficient (PCC). In addition, we studied the effects of DRTs, model complexity, and sample size on the predictive performance of SDMs. The predictive performance of SDMs under DRTs other than KPCA is better than using PCC. And the predictive performance of SDMs using linear DRTs is better than using nonlinear DRTs. In addition, using DRTs to deal with environmental variables has no less impact on the predictive performance of SDMs than model complexity and sample size. When the model complexity is at the complex level, PCA can improve the predictive performance of SDMs the most by 2.55% compared with PCC. At the middle level of sample size, the PCA improved the predictive performance of SDMs by 2.68% compared with the PCC. Our study demonstrates that DRTs have a significant effect on the predictive performance of SDMs. Specifically, linear DRTs, especially PCA, are more effective at improving model predictive performance under relatively complex model complexity or large sample sizes.

## Graphical abstract

Dimensionality reduction techniques (DRTs) can effectively improve the predictive performance of species distribution models by reducing the dimensionality of environmental variables. Specifically, linear DRTs (especially principal component analysis, or PCA) were found to be more effective in improving model performance under relatively complex model complexity or large sample sizes.

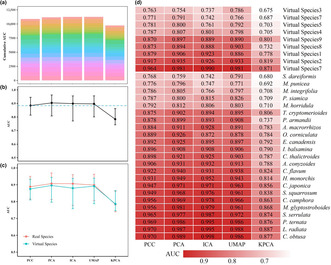

## 1 INTRODUCTION

Effective prediction of species distribution is crucial for ecologists studying various ecological issues such as species diversity (Allouche et al., 2006; Hao et al., 2019; Norberg et al., 2019), conservation (Maiorano et al., 2019; Wang et al., 2021; Whitehead et al., 2014), and biological invasions (Chapman et al., 2019). The potential distribution of species can be determined by the known geographical locations of species, environmental conditions (e.g., bioclimatic variables), and other factors (such as biotic interactions) (Acevedo et al., 2012; Soberón, 2010). Species distribution models (SDMs), which provide a mathematical framework for expressing the relationship between species location and environment, are increasingly widely used by ecologists to predict the potential distribution of species (Acevedo et al., 2012; Hao et al., 2019). And the factors affecting the accuracy of prediction, such as algorithms, samples, and environmental variables, have been extensively studied (Iturbide et al., 2015; Li & Wang, 2013; Liang et al., 2018; Liu et al., 2019; Naimi et al., 2011; Van Eupen et al., 2021). Among the commonly applied algorithms for SDMs are maximum entropy (Bradie & Leung, 2017; Li et al., 2020), random forest (Behera et al., 2021; Bradter et al., 2013), support vector machines (Muñoz‐Mas et al., 2016), and even the ensemble of multiple algorithms (Grenouillet et al., 2011; Hao et al., 2019). While research on algorithms for predicting species distribution has been extensive, few new algorithms have been proposed in recent years that improve upon existing ones. In addition to algorithms, the influence of sample points, such as sampling bias (Bean et al., 2012; Syfert et al., 2013) and spatial autocorrelation (Guélat & Kéry, 2018), on the prediction accuracy of SDMs has been widely examined.

Previous research on environmental variables in SDMs has focused on how to select variables to deal with collinearity problems (such as using pairwise Pearson correlation coefficients or variance inflation factor analysis) that can lead to inaccurate interpretation of results by variables or uncertainty in model fit (Cobos et al., 2019; De Marco & Nóbrega, 2018; Dormann et al., 2013; Maiorano et al., 2019; Shi et al., 2019). With more and more environmental variables available, a remaining challenge for SDMs is how to take advantage of massive environmental variable datasets. Dimensionality reduction techniques (DRTs), including both linear and nonlinear, have been proposed as an effective solution to this problem (Ayesha et al., 2020; De Marco & Nóbrega, 2018; Dormann et al., 2013; Meng et al., 2016; Reddy et al., 2020). Linear DRTs have the advantage of requiring less computation power, while nonlinear DRTs may have high computational time and cost but are also successfully used for feature extraction of complex data such as biomedical, audio, and video (Ayesha et al., 2020; Fodor, 2002; Reddy et al., 2020; Van Der Maaten et al., 2009). DRTs can transform high‐dimensional datasets into low‐dimensional ones while retaining most of the variance explained by the data (Juvonen et al., 2015) and reducing computation time and storage space requirements (Ayesha et al., 2020; Lesort et al., 2018; Meng et al., 2016; Verleysen & François, 2005). Some studies have shown that using DRTs for variable dimension reduction can tremendously reduce the time and complexity for the training phase of machine learning algorithms (Ayesha et al., 2020; Reddy et al., 2020) and also improve their predictive performance (Cha et al., 2021; De Marco & Nóbrega, 2018; Merow et al., 2014; Reddy et al., 2020; Vignali et al., 2020). Given that most SDMs are built based on machine learning algorithms, we hypothesize that incorporating DRTs to process environmental variables in SDMs can improve their predictive performance.

Despite their potential benefits, DRTs for environmental predictors other than principal component analysis (PCA) (De Marco & Nóbrega, 2018; Dupin et al., 2011; Hanspach et al., 2011; Norberg et al., 2019; Silva et al., 2014; Velazco et al., 2017; Wellmann et al., 2020) have been rarely used for SDMs. There are many other DRTs, such as linear discriminant analysis (LDA) (Press & Wilson, 1978; Tharwat et al., 2017), independent component analysis (ICA) (Comon, 1994), isometric mapping (ISOMAP) (Lee et al., 2004), locally linear embedding (LLE) (Roweis & Saul, 2000), kernel principal component analysis (KPCA) (Schölkopf et al., 1997), and uniform manifold approximation and projection (UMAP) (Becht et al., 2019; Mcinnes & Healy, 2018). These DRTs have been successfully applied in various fields, such as image and speech processing, data analysis and compression, source localization, document classification, and cluster analysis (Ayesha et al., 2020; Jolliffe & Cadima, 2016; Meng et al., 2016). However, various DRTs have been developed to solve different types of issues, as LDA was proposed to determine the subject of text data (Fisher, 1936) and ICA was proposed to separate mixed signals (Comon, 1994). That is to say, not all DRTs are suitable for environmental variables dimensionality reduction in SDMs. Therefore, there is a need to thoroughly explore the use of DRTs for environmental variables in SDMs to improve their predictive performance. This would not only benefit SDM researchers but also help advance our understanding of species–environment relationships.

Furthermore, previous studies have shown that model complexity (Brun et al., 2020; Werkowska et al., 2017) and sample size (Bean et al., 2012; Liu et al., 2019; Stockwell & Peterson, 2002; Van Proosdij et al., 2016; Wisz et al., 2008) can influence the predictive performance of SDMs; that is, intermediate to more complex model complexity (Brun et al., 2020) and larger sample sizes (Wisz et al., 2008) can result in better performance. The model complexity here refers to adjusting the level of complexity within SDM algorithms by modifying a set of parameters. However, it is unclear whether using DRTs in combination with varying levels of model complexity or sample size can further improve the predictive performance of SDMs. Hence, further investigation is needed to determine the appropriate level of model complexity or sample size when using DRTs to process environmental variables in SDM.

In this study, we used 45 environmental variables (such as bioclimatic variables, terrain variables, and soil variables) to construct SDMs and investigated the impact of using DRTs on the predictive performances of SDMs compared to the commonly used method of selecting environmental variables based on Pearson's correlation coefficient (PCC). This number of environmental variables exceeds that typically used for SDMs studies. Predictive performance here refers to the accuracy of the model that has been validated by the test set. Specifically, we applied linear and nonlinear DRTs to reduce the dimensionality of high‐dimensional environmental variables and established SDMs based on the resulting low‐dimensional dataset. We then evaluated the predictive performance of more than 70,000 SDMs (32 species × 5 data preprocess × 5 SDMs × 3 model complexity × 30 replicates) using three evaluation metrics. Additionally, we examined the contribution of DRTs, sample size, and model complexity to the performance of SDMs and explored the appropriate level of model complexity and sample size for using DRTs on environmental variables. Our analysis sheds light on whether DRTs can significantly enhance the predictive performance of SDMs and informs the development of more effective modeling approaches for SDMs.

## 2 METHODS

### 2.1 Overview

**FIGURE 1:**
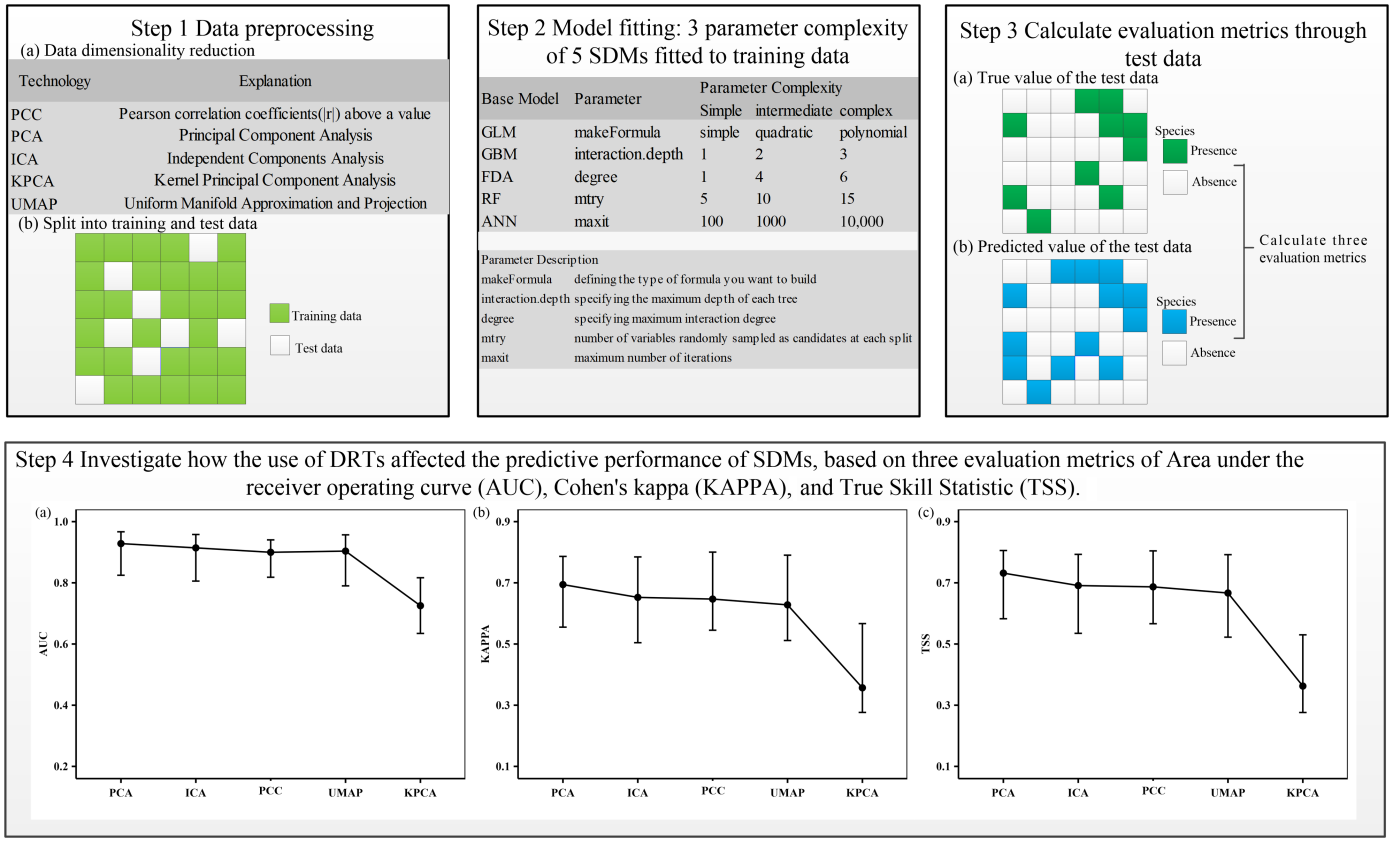
The workflow of this study. We preprocessed the data (Step 1), fitted models based on training data (Step 2), calculated three evaluation metrics based on the true and predicted values of the test data (Step 3), and investigated the influence of dimensionality reduction techniques (DRTs) on the predictive performance of species distribution models (SDMs) from the metrics of area under the receiver operating characteristic curve (AUC), Cohen's kappa (KAPPA), and the true skill statistic (TSS) (Step 4).

Here, we used the four commonly used DRTs to investigate their impact on the performance of five SDM algorithms. The four DRTs included PCA, independent components analysis (ICA), KPCA, and UMAP, while the five SDM algorithms were generalized linear models (GLMs), generalized boosted models (GBMs), flexible discriminant analysis (FDA), random forests (RF), and artificial neural networks (ANN). Our dataset included a pool of environmental variables corresponding to the occurrence records of 32 species. Four primary steps were conducted to implement this study. First, in the preprocessing phase, we applied the four DRTs to the environmental variables dataset and obtained the most prominent components of the environmental variables, respectively. We extracted the data corresponding to the species occurrence records from the environmental variables dataset after dimensionality reduction, and then randomly selected 80% of them as the training set for training the model and the remaining 20% as the test set for evaluating the model, and repeated the same procedure for 30 replicates for a species. Second, for each set of training data for a species, we fitted the five SDM algorithms at three levels of parameterized complexity. Third, we calculated three evaluation metrics for evaluating the predictive performance of SDMs based on the true and predicted values of the test data. Fourth, we investigated how the use of DRTs affected the predictive performance of SDMs. In addition, we compared the model prediction results constructed based on the new environmental variables selected through PCC with those of the DRTs to conduct a comparative study. The general workflow of our study is summarized in Figure 1.

### 2.2 Data

#### 2.2.1 Species occurrence data

We first obtained occurrence records for 23 plant species from the Global Biodiversity Information Facility (GBIF, http://www.gbif.org/; the complete list of data sources is given in Table S2) in order to perform this investigation using plants. We selected the plants based on their habitats (e.g., terrestrial and aquatic), range size (e.g., global, continental, and local), climate zone (e.g., tropical and temperate), altitude (e.g., alpine and subalpine), plant type (e.g., annual herb and perennial herb), and habit (e.g., thermophilic, ombrophyte, and hygrophilo) in order to include as many different characteristics of plants as we could (the detailed characteristics of each plant species in Table S2). We only downloaded occurrence records collected after 1970 and removed duplicate records within a 5‐km radius. The resulting dataset contained between 51 and 7007 records per species (see Figures S1 and S2 for the distribution of species' occurrence records and Table S2 for the species basic information in Data  S1). The pseudo‐absence data per species were randomly taken from the study area according to three times the amount of the occurrence records data (e.g., 100 occurrence records data, 300 pseudo‐absence data). And we extended the range of the occurrence records outward by 1° as the study area for each species (Phillips & Dudík, 2008). We categorized the 23 species into three kinds of sample sizes according to the number of occurrence records and calculated the prevalence as the ratio of the number of occupied cells of each species to the number of cells within its geographical distribution extent (De Marco & Nóbrega, 2018). Species with less than 100 occurrence records were classified as having a small sample size (Small); those between 100 and 1000 records were classified as having a middle sample size (Middle); and those with over 1000 records were classified as having a large sample size (Big).

To overcome the influence of sampling bias, spatial autocorrelation, and other problems and to facilitate comparison with real species distributions (De Marco & Nóbrega, 2018), we further followed Leroy et al. (2016) to generate geographic distributions of the nine virtual species via the R package “virtualspecies”. We first determined the distribution range and prevalence of 23 real species according to their occurrence records and then cross‐selected the distribution range and prevalence (see Table S1 for the prevalence of each species) to generate nine virtual species, which basically covered the distribution characteristics of 23 real species. To generate virtual species, we performed four steps: (1) generating the virtual species' environmental suitability using the function “generateRandomSp”, (2) converting the environmental suitability into presence–absence with the function “convertToPA”, (3) limiting the species distribution using the function “limitDistribution”, and (4) sampling observed occurrences for the virtual species using the function “sampleOccurrences”. The prevalence is used in the second step of generating virtual species. The virtual species' names and occurrence records are listed in Table S1.

#### 2.2.2 Environmental data

We first downloaded 19 bioclimatic variables of the current climate (average of 1970–2000) at a resolution of 2.5 arc‐min and elevation data at a resolution of 30 arc‐sec from WorldClim 2.1 (http://www.worldclim.org/) (Fick & Hijmans, 2017). Then, we derived terrain ruggedness (maximum elevation difference in each 5 × 5 km^2^ cell), topographic position index (difference between the elevation of a cell and the mean value of its 24 surrounding cells; each cell is 1 × 1 km^2^), mean slope, maximum difference of slope in each 5 × 5 km^2^ cell, and aspect, with a resolution of 2.5 arc‐min, from the elevation data. Third, 18 soil variables were obtained from the Harmonized World Soil Database (HWSD) with a spatial resolution of 30 arc‐sec (FAO et al., 2012). And data representing ecological indicators, including potential evapotranspiration (PET) and the aridity index (AI), were acquired at a resolution of 30 arc‐sec from Version 3 of the Global Aridity Index and Potential Evapotranspiration Database (Zomer et al., 2022). Finally, we obtained 45 environmental variables and resampled all of them with a resolution of 2.5 arc‐min. Abbreviations and full names of all environmental variables were listed in Table S3.

### 2.3 DRTs

In this section, we briefly introduce two linear and two nonlinear DRTs and how we used the above‐mentioned DRTs for environmental variables in this study. We implemented all our DRTs in the R environment (version 4.1.1, R Core Team, 2021).

#### 2.3.1 Linear DRTs

PCA is an unsupervised linear mapping based on eigenvector search that converts a set of correlated variables into a set of uncorrelated variables, usually with the user setting retaining a set of components that explain at least 95% of the total variance (i.e., fixed cumulative eigenvalue criteria) as the selected components (axes) (Abdi & Williams, 2010). We implemented PCA with the R package “stats” (version 4.0.5). Independent component analysis (ICA) is an unsupervised linear DRT (Comon, 1994) to extract independent components from a set of linear transformations of the original data. ICA finds a linear mapping of the source vector such that each component of an estimate is as independent as possible and often selects components (axes) based on subjectivity, that is, specifying the number of independent components to be selected (Ayesha et al., 2020; Pham & Garat, 1997). We implemented ICA with the R package “fastICA” (version 1.2‐3).

#### 2.3.2 Nonlinear DRTs

As an extension of conventional PCA, KPCA estimates the covariance matrix of the new feature vectors after transforming the input data into kernel space (Ayesha et al., 2020; Schölkopf et al., 1997). We implemented KPCA with the R package “kernlab” (version 0.9‐30). In contrast, UMAP is a topology‐based approach that constructs low‐dimensional representations of high‐dimensional data by approximating the local manifold structure. The topology‐based approach is an approach that preserves the topology structure of the data. Topology structure describes the proximity (that is, which data points are adjacent or close) and the connectivity (that is, which data points are connected together) between data points, as well as the cluster structure of data points (that is, which data points belong to the same cluster). Manifold structure is the internal geometry of data in high‐dimensional space, which can describe the relationship between data points and the local continuity between each other. UMAP constructs a weighted k‐nearest neighbor graph of the input data before computing the low‐dimensional layout of the graph (Mcinnes et al., 2018; Mcinnes & Healy, 2018). We implemented UMAP with the R package “umap” (version 0.2.8.0).

We aim to reveal whether DRTs are superior to PCC and whether DRTs have an impact on the predictive performance of SDMs in this study. However, there is no definitive conclusion on how many components (axes) should be selected for analysis in the process of dimensionality reduction (Ayesha et al., 2020). For this reason, we selected the same number of components (axes) for each DRT as for PCA, where the components (axes) selected for PCA capture 95% of the variance explained. To compare the effects of different DRTs on SDMs, we included the most commonly used PCC as the control. We first retained three sets of compliant environment variables based on three criteria (PCC < 0.70, <0.75, and <0.80). Then, the three selected sets of variables are applied to the model, respectively. Finally, we selected the variables under the criterion that led to the best predictive performance of the model, as measured by AUC (Table S4). The variables selected through PCC for each species were listed in Table S5.

### 2.4 Species distribution modeling

We fitted five SDM algorithms with three levels of model complexity: simple, intermediate, and complex, respectively. The SDM algorithms included two regression techniques (i.e., GLM and FDA), two tree‐based techniques (i.e., RF and GBM), and a heuristic algorithm (i.e., ANN). We set the basic parameters of each SDM as follows: For GLM, we set the binomial distribution with a logit link function, a horizontal depth of interaction between explanatory variables of one (i.e., at most two explanatory variables generate interaction terms), and the optimal model was determined by stepwise regression (stepwise search direction is both) according to the Akaike information criterion (AIC) value via the R package “stats” (version 4.0.5). We set the method as multivariate adaptive regression splines for FDA via the R package “mda” (version 0.5‐3), the number of trees to grow as 1000, and the minimum size of terminal nodes as 20 for RF via the R package “randomForest” (version 4.6‐14), and the total number of trees as 1000 and a shrinkage parameter applied to each tree in the expansion as 0.01 for GBM via the R package “gbm” (version 2.1.8). Furthermore, in ANN, we set initial random weights at [−0.1, 0.1] and used cross‐validation (the cross‐validation here is a procedure that runs inside ANN, not in the dataset processing phase) to select the optimal size of the hidden layer and weight decay via the R package “nnet” (version 7.3‐17).

We set the model complexity of SDMs by adjusting the flexibility of the response curves in GLM, the maximum interaction degree in FDA, the maximum number of iterations in ANN, the number of variables randomly sampled as candidates at each split in RF, and the maximum depth of each tree in GBM, respectively. For GLM, we used an intercept term and linear terms of predictor variables as the simple model, added quadratic terms to the simple model as the intermediate model, and added third‐order polynomials to the intermediate model as the complex model. For FDA, we set the maximum interaction degree to 1, 4, and 10, representing simple, intermediate, and complex parameterizations, respectively. We also adjusted the maximum number of iterations to 100, 1000, and 10,000 for ANN complexity.

For RF and GBM, we set up two sets of comparison experiments of important parameters to determine the parameters considered for the final model complexity, respectively (see Figure S11 for details). In RF, we used the number of variables randomly sampled as candidates at each split to tune model complexity (values 5, 10, and 15), where larger values mean more variables sampled per split. In GBM, we used the maximum depth of each tree to tune model complexity (values 1, 2, and 3), where larger values mean more variable interactions.

### 2.5 Model evaluation metrics

Studies have shown that using only one evaluation metric to measure the predictive performance of SDMs will lead to misleading conclusions (Yu et al., 2020). In addition, different evaluation metrics have their own characteristics. Area under the receiver operating characteristic curve (AUC) is threshold‐independent, while Cohen's kappa (KAPPA) and TSS rely on thresholds, but TSS is unaffected by prevalence (Allouche et al., 2006). For this reason, three metrics were selected in this study. The first metric, AUC, measures the ability of the model to distinguish between the presence and absence of the species. AUC values range between 0 and 1, with a value closer to 1 indicating a good model fit for predicting species distributions (Manel et al., 2001; Swets, 1988). The second metric is KAPPA, with a value between −1 and 1. A higher KAPPA value indicates better model predictions (Cohen, 1960; Pearson et al., 2004). The third metric is the true skill statistic (TSS), which is equal to the sum of sensitivity and specificity minus one (Allouche et al., 2006). TSS values range from −1 to 1, with a value closer to 1 indicating a good model fit for predicting species distributions. In this study, we used a threshold value at which the TSS is maximized to determine presences and absences. We compared the predictive performance of GLM, GBM, FDA, RF, and ANN models through AUC, KAPPA, and TSS as evaluation metrics, as summarized in Table 1.

**TABLE 1:** Descriptions, ranges, and criteria for metrics used to evaluate model predictive performance.

Metric	Description	Range	Criterion
AUC	Area under the curve of the receiver operating characteristic (ROC)	0 ~ 1	Closer to 1
KAPPA	Measurement of inter‐rater reliability	−1 ~ 1	>0.2
TSS	The true skill statistic: sensitivity + specificity −1	−1 ~ 1	Closer to 1

### 2.6 Analysis of the impact of DRTs on the predictive performance of SDMs

We trained all models using a random 80% sample of species data, leaving the remaining 20% for model evaluation, and repeated the same procedure for 30 replicates. For each replicate, we evaluated the predictive performance of each model through AUC, KAPPA, and TSS. All the analyses described below are based on the median of evaluation metrics, which are not affected by extreme values and better represent the true predictive performance of the model. To investigate the impact of DRT on SDMs, we first compared the predictive performance of SDMs using DRTs with that of SDMs using PCC based on model evaluation metrics (i.e., AUC, KAPPA, and TSS). Then, we quantified the relative contributions of SDM algorithms, DRTs, sample size, and model complexity using model evaluation metrics and conducted a multivariate analysis of variance (MANOVA) to assess the significance of their influence on model performance. We also categorized model complexity into complex, intermediate, and simple (see Section 2.4 for complexity setting) and sample size into big, middle, and small (see Table S1 for species classification). To account for the interaction effects of SDM algorithms, model complexity, sample size, and DRTs, we also examined the significance of their linear interactions on model performance. We further analyzed the effect of DRTs on model performance after SDMs were subjected to different levels of model complexity or sample size and identified the level of model complexity or sample size combined with DRTs that could improve model prediction performance more effectively. Additionally, we conducted a statistical analysis of the predicted results for real and virtual species to verify the consistency of the above results.

## 3 RESULTS

### 3.1 The effect of DRTs on SDMs performance

**FIGURE 2:**
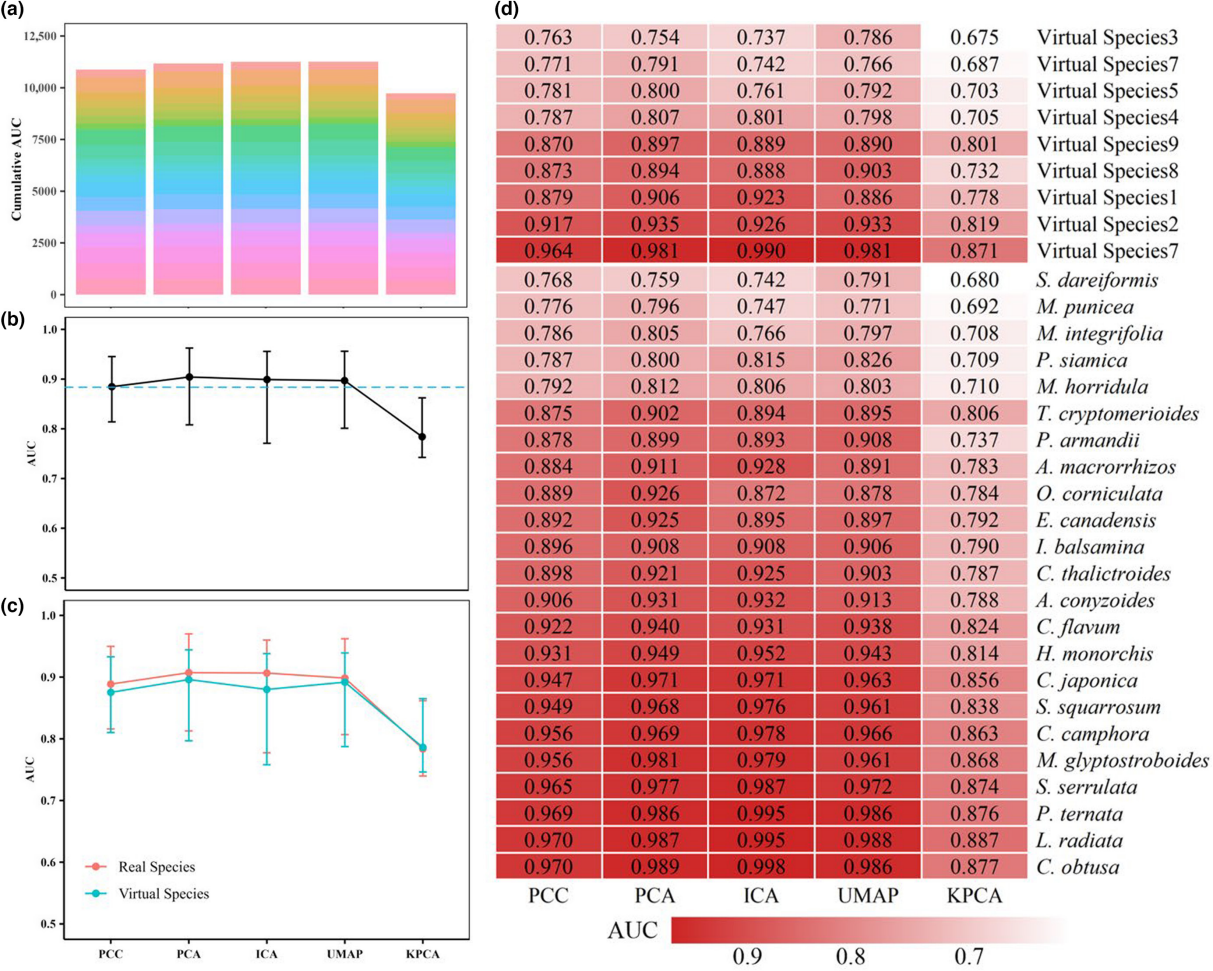
Statistical results of the predictive performance (measured by AUC) of five SDMs (i.e., GLM, GBM, RF, FDA, and ANN) constructed after the environmental variables were treated with the four DRTs (i.e., PCA, ICA, UMAP, and KPCA) and PCC. (a) The cumulative AUC value for all SDMs predicting 32 species distributions under each DRT and PCC. (b) The median of AUC values for all SDMs predicting 32 species distributions under each DRT and PCC. (c) The median of AUC values for all SDMs predicting the species distribution of real and virtual species under each DRT and PCC. (d) The median of AUC values for all SDMs predicting species distribution of each species under each DRT and PCC; the row and the column represent the median of the prediction results for different species under four DRTs and PCC, respectively. See Table S1 for specific species names. ANN, artificial neural networks; AUC, area under the receiver operating characteristic curve; DRTs, dimensionality reduction techniques; FDA, flexible discriminant analysis; GBM, generalized boosted model; GLM, generalized linear model; ICA, independent component analysis; KPCA, kernel principal component analysis; PCA, principal component analysis; PCC, Pearson's correlation coefficient; RF, random forests; SDMs, species distribution models; UMAP, uniform manifold approximation and projection.

**FIGURE 3:**
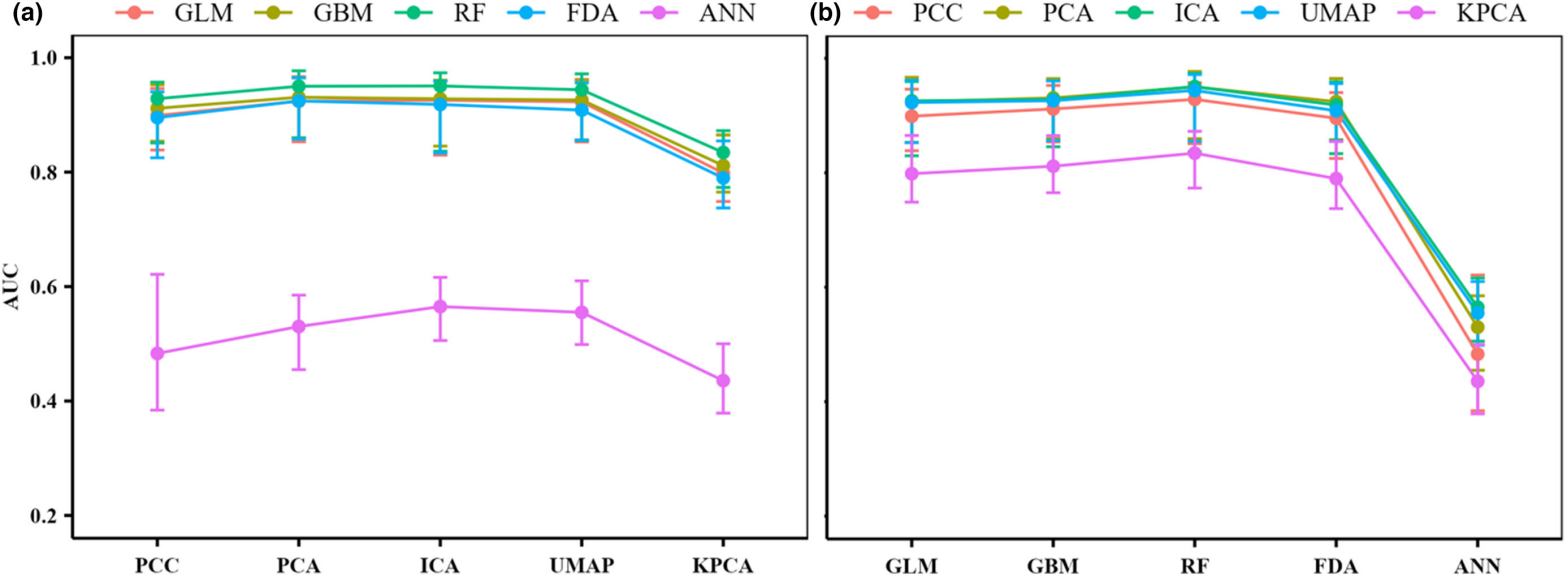
Statistical results of predictive performance (measured by AUC) of five species SDMs (i.e., GLM, GBM, RF, FDA, and ANN) constructed after the environmental variables were treated with the four DRTs (i.e., PCA, ICA, UMAP, and KPCA) and PCC. (a) The median of AUC values for different SDMs predicting 32 species distributions under each DRT and PCC. (b) The median of AUC values for each SDM predicting 32 species distributions under different DRTs and PCC. ANN, artificial neural networks; AUC, area under the receiver operating characteristic curve; DRTs, dimensionality reduction techniques; FDA, flexible discriminant analysis; GBM, generalized boosted model; GLM, generalized linear model; ICA, independent component analysis; KPCA, kernel principal component analysis; PCA, principal component analysis; PCC, Pearson's correlation coefficient; RF, random forests; SDMs, species distribution models; UMAP, uniform manifold approximation and projection.

The predictive performance of SDMs under DRTs other than KPCA is better than using PCC (Figures 2, S3 and S4, and Tables 2, S6 and S7). And the predictive performance of SDMs using the linear DRTs (i.e., PCA and ICA) is better than that of those using the nonlinear DRTs (i.e., UMAP and KPCA) (Figures 2, S3 and S4). The effects of DRTs on SDMs of 23 real species and nine virtual species show high consistency with the above results (Figures 2c,d, S3 and S4). In addition, the responses of GLM, GBM, RF, and FDA to four DRTs and PCC are consistent (Figures 3a, S3 and S4), and PCA is most suitable for the above four SDMs (Figures 3b, S3b and S4b, and Tables 2, S6 and S7). Although the overall prediction performance of ANN is not good, ANN after dimensionality reduction can indeed improve the prediction performance compared to PCC (Figures 3a, S3a, S4a, and Tables 2, S6 and S7). In addition, DRTs improve the predictive performance of ANN much more than those of the other four SDMs (Tables 2, S6 and S7). And the top three with the best DRTs are PCA, ICA, and UMAP, and KPCA is the worst (Figures 3b, S5b and S6b).

**TABLE 2:** The percentage improvement in species distribution models (SDMs, i.e., GLM, GBM, FDA, RF, and ANN) predictive performance (measured by AUC) using four dimensionality reduction techniques (DRTs, i.e., PCA, ICA, UMAP, and KPCA) compared with using Pearson's correlation coefficient (PCC).

	GLM	GBM	FDA	RF	ANN
PCA‐PCC	2.79%	2.11%	3.24%	2.34%	9.74%
ICA‐PCC	2.96%	1.82%	2.57%	2.40%	16.94%
UMAP‐PCC	2.66%	1.59%	1.46%	1.68%	14.87%
KPCA‐PCC	−11.18%	−10.98%	−11.78%	−10.13%	−9.84%

### 3.2 Analysis of significant factors affecting predictive performance

**FIGURE 4:**
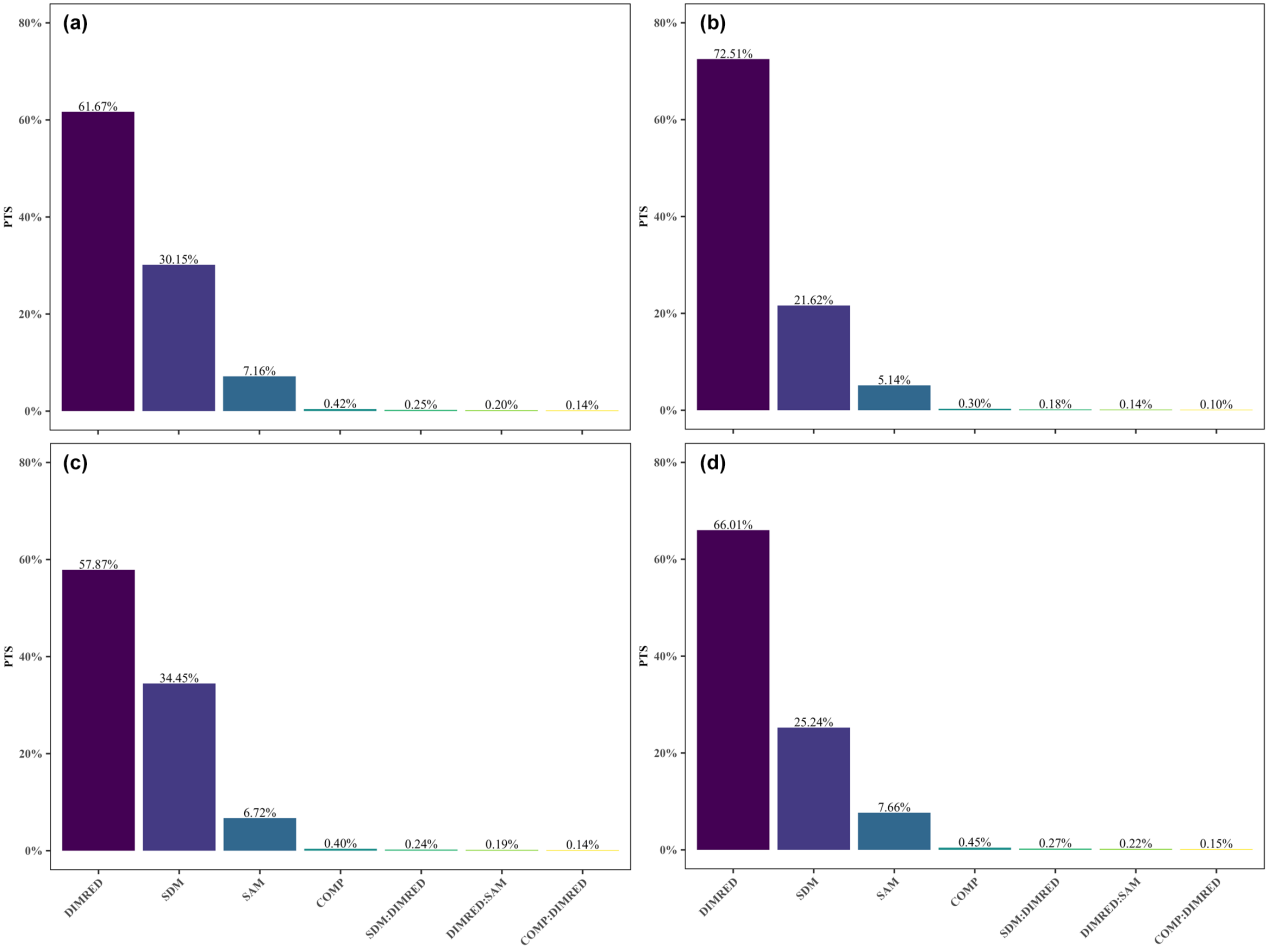
Relative contributions of different influencing factor on predictive performance assessed by MANOVA, and the evaluation metric is the AUC. SDM and DRT among the influencing factors, considers four scenarios: (a) including all SDMs (i.e., GLM, GBM, RF, FDA, ANN) and DRTs (i.e., PCA, ICA, UMAP, KPCA) considered in this study, (b) ANN in SDMs is removed, (c) KPCA in DRTs is removed, and (d) ANN in SDMs and KPCA in DRTs are both removed. Bars represent the PTS. The bars labeled “COMP,” “DIMRED,” “SDM,” and “SAM,” respectively, represent model complexity, dimensionality reduction, the SDM algorithm, and sample size. The bars labeled “COMP:DIMRED,” “SAM:DIMRED,” and “SDM:DIMRED,” respectively, represent the interaction between model complexity and dimensionality reduction, the interaction between sample size and dimensionality reduction, as well as the interaction between the SDM algorithm and dimensionality reduction. ANN, artificial neural networks; AUC, area under the receiver operating characteristic curve; DRTs, dimensionality reduction techniques; FDA, flexible discriminant analysis; GBM, generalized boosted model; GLM, generalized linear model; ICA, independent component analysis; KPCA, kernel principal component analysis; MANOVA, multivariate analysis of variance; PCA, principal component analysis; PTS, proportion of total sums of squares; RF, random forests; SDMs, species distribution models; UMAP, uniform manifold approximation and projection.

When all SDMs and DRTs were considered, DRTs had a significant influence on species distribution prediction from the evaluation metrics of AUC, KAPPA, and TSS (Table 3 and Figures 4a, S7a and S8a), which correspond to the highest proportion of total sum of squares (PTS) with about 61.67% when the contribution of the factor to the model is measured by the increment of AUC (Figure 4a). PTS is expressed as the ratio of the squares of the contribution of the influencing factor for species distribution prediction to the total sum of the squares of the contribution of all factors. In other words, DRTs were the most important influence factor for the predictive performance of SDMs (Figures 4a, S7a and S8a) in the case considered in this study. The second and third most important factors were SDM algorithms and sample size, with PTS of 30.15% and 7.16%, respectively (Figure 4a). The contributions of model complexity were relatively less important (PTS = 0.42%). In addition, the interaction effects of model complexity, sample size, the SDM algorithm, and DRTs were very small, with the maximum PTS not exceeding 1% (Figures 4a, S7a and S8a).

**TABLE 3:** Results of MANOVA for area under the receiver operating characteristic curve (AUC), Cohen's kappa (KAPPA), and true skill statistic (TSS)

	DF	AUC		KAPPA		TSS	
		*F* value	Pr (>*F*)	*F* value	Pr (>*F*)	*F* value	Pr (>*F*)
DIMRED	4	4694.71***	<2e‐16	4345.68***	<2e‐16	5663.12***	<2e‐16
SAM	2	544.939	<2e‐16	335.34	<2e‐16	782.45	<2e‐16
SDM	3	2294.82	<2e‐16	1898.80	<2e‐16	2348.69	<2e‐16
COMP	2	32.28	<2e‐16	50.77	<2e‐16	77.88	<2e‐16
SDM:DIMRED	8	25.31	.005	38.21	<2e‐16	53.83	<2e‐16
SAM:DIMRED	12	19.11	1.33e‐12	23.32	<2e‐16	28.74	<2e‐16
COMP:DIMRED	8	11.01	1.52e‐14	10.56	.00345	12.55	.001279

*Note*: “DIMRED,” “SAM,” “SDM,” and “COMP,” respectively, represent dimensionality reduction, sample size, the SDM algorithm, and model complexity. “SAM:DIMRED,” “SDM:DIMRED,” and “COMP:DIMRED,” respectively, represent the interaction between sample size and dimensionality reduction, the interaction between the SDM algorithm and dimensionality reduction, as well as the interaction between model complexity and dimensionality reduction. Significant are denoted by asterisks (*p < .05, **p < .01, and ***p < .001).

Abbreviation: DF, degrees of freedom.

The predictive performance of KPCA in DRTs, however, is much lower than that of the other DRTs, while the predictive performance of ANN in SDMs is significantly lower than that of the other SDMs, as shown in Figure 3. We took into account the following three scenarios to further verify whether the above conclusions are brought on by KPCA or ANN. When ANN in SDMs was removed, DRTs were still the most important influence factor for the predictive performance of SDMs; SDM algorithms were second, but there was a decrease in the relative contribution of SDM algorithms (from 30.15% to 21.62%) (Figure 4a,b). Similarly, when KPCA in DRTs was removed, DRTs still remained the most important influence factor for the predictive performance of SDMs, even though the relative contribution of DRTs was reduced (from 61.67% to 57.87%) (Figure 4a,c). When ANN in SDMs and KPCA in DRTs were removed, the relative contribution of DRTs increased (from 61.67% to 66.01%) while the relative contribution of SDMs decreased (from 30.15% to 25.24%) (Figure 4a,d), and DRTs remained the most important factor affecting the predictive performance of SDMs. We considered the latter three scenarios separately to prevent KPCA in DRTs and ANN in SDMs from leading to misleading conclusions.

### 3.3 The interaction of DRTs, model complexity, and sample size on the predictive performance of SDMs

**FIGURE 5:**
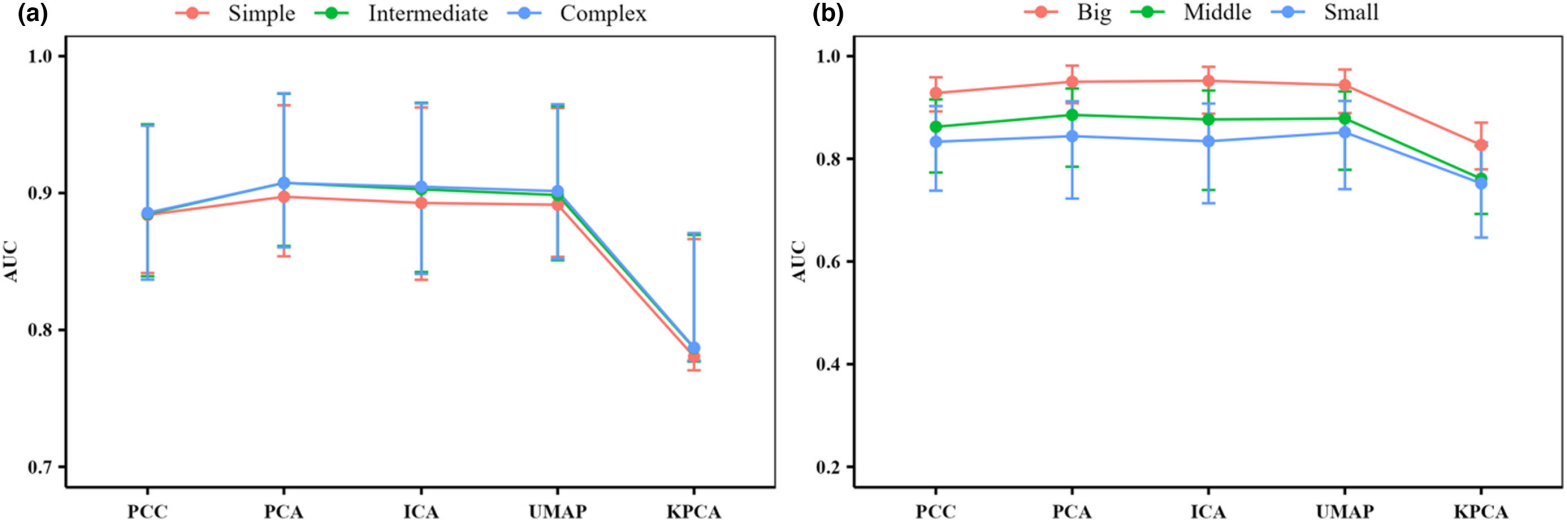
Statistical results of the predictive performance (measured by AUC) of SDMs (i.e., GLM, GBM, RF, FDA, and ANN) under different levels of model complexity (simple, intermediate, and complex) and sample size (small, middle, and big). (a) The median of AUC values for all SDMs, based on different model complexity, predicts 32 species distributions under each DRT and PCC. (b) The median of AUC values for all SDMs, based on different sample sizes predicts 32 species distributions under each DRT and PCC. Based on the sample size (occurrence records) of species, the species are categorized into three groups. Species with less than 100 occurrence records were classified as small; those between 100 and 1000 records were classified as middle; and those with over 1000 records were classified as big. ANN, artificial neural networks; AUC, area under the receiver operating characteristic curve; DRT, dimensionality reduction technique; FDA, flexible discriminant analysis; GBM, generalized boosted model; GLM, generalized linear model; PCC, Pearson's correlation coefficient; RF, random forests; SDMs, species distribution models.

SDMs with intermediate or complex parameters performed better for prediction than simple parameters when DRTs were used (Figures 5a, S9a and S10a). In contrast, when the environmental variables were processed through PCC, model complexity did not significantly affect the predicted performance of SDMs (Figures 5a, S9a and S10a). More specifically, the predictive performance of SDMs improved with increasing model complexity when combined with DRTs (Figures 5a, S9a and S10a, and Tables 4, S8 and S9). For the complex level of model complexity, the predictive performance of SDMs was improved more when applying PCA, ICA, and UMAP to the environment variables than when applying PCC. Among them, the improvement was the largest when PCA was used, reaching 2.55% (Figures 5a, S9a and S10a, and Table 4). For the sample size, the predictive performance increased with increasing sample size when DRTs or PCC were used (Figures 5b, S9b and S10b). Different levels of sample size combined with DRTs (or PCC) lead to slight differences in the improvement of the predictive performance of SDMs. SDMs with a middle or big sample size performed better for prediction than those with a small sample size when linear DRTs (i.e., PCA and ICA) were used (Figures 5a, S9a and S10a). In particular, for the middle level of sample size, the predictive performance of SDMs when PCA was applied to the environment variables improved the most compared with that when PCC was applied, reaching 2.68% (Figures 5b, S9b and S10b, and Table 4). For the big level of sample size, PCA, ICA, and UMAP in DRTs outperform PCC in model performance (Figures 5b, S9b and S10b, and Tables 4, S6 and S7).

**TABLE 4:** The percentage improvement in SDMs predictive performance (measured by AUC) using different dimensionality reduction techniques (i.e., PCA, ICA, UMAP, and KPCA) and Pearson's correlation coefficient (PCC) under different model complexity (i.e., simple, intermediate, and complex) or sample size (i.e., small, middle, and big).

	Model complexity			Sample size		
	Simple	Intermediate	Complex	Small	Middle	Big
PCA‐PCC	1.50%	2.46%	2.55%	1.30%	2.68%	2.37%
ICA‐PCC	0.99%	2.02%	2.15%	0.11%	1.65%	2.56%
UMAP‐PCC	0.85%	1.55%	1.79%	2.20%	1.87%	1.66%
KPCA‐PCC	−11.70%	−11.09%	−11.12%	−9.74%	−11.74%	−10.91%

## 4 DISCUSSION

Our study found that using DRTs to preprocess environmental variables generally has a positive effect on the performance of SDMs, outperforming the traditional PCC method, though this effect can be relatively small. In addition, this effect may increase when other evaluation methods are used, such as block cross‐validation, which more directly tests model transferability (Roberts et al., 2017), and future work should examine this. Specifically, SDMs combined with linear DRTs such as PCA and ICA demonstrate higher values of performance metrics such as AUC, KAPPA, and TSS, probably as both methods generate new variables by combining the original variables in a linear manner, which allows for a reduction in dimensionality without significant loss of information. In addition, using DRTs to process variables can remove redundant features among variables, retain the most useful information, and avoid the risk of excluding highly correlated but important variables when using PCC to screen variables (Ayesha et al., 2020; Reddy et al., 2020). Our research thus recommends using PCA or ICA to reduce the dimensionality of environmental variables for SDMs.

Our analysis indicates that the use of DRTs has a greater impact on SDMs performance compared to model complexity and sample size. The reason for being significant is that the model complexity and sample size we have set are already appropriate; that is, the complexity of the model we selected is not based on relatively fewer parameters and fewer predictors of the relationship, and the sample size we selected is greater than 30. A simple model may lack the flexibility to accurately describe the complex relationship between environmental factors and species distribution (Werkowska et al., 2017), leading to a potential misinterpretation of the underlying factors that drive species distribution (Wisz et al., 2008). Therefore, we think that the impact of DRT is no less than that of model complexity and sample size, at least for the plants selected for this study. Even under reasonable model complexity and sample size, the impact of DRT on SDMs is more significant.

SDMs with intermediate or complex model complexity perform well, which is in line with earlier studies showing that models fitted with relatively complex parameterizations will perform better (Chala et al., 2016; Gregr et al., 2019). Our findings also support past research showing that the predictive performance of SDMs increases with an increase in sample size (Bean et al., 2012; Liu et al., 2019; Stockwell & Peterson, 2002; Van Proosdij et al., 2016; Wisz et al., 2008). Furthermore, different levels of model complexity or sample size combined with DRTs used to process environment variables will lead to differences in the improvement of SDMs predictive performance. Specifically, linear DRTs (especially PCA) are more effective at improving model performance under relatively complex model complexity or large sample sizes. For SDM applications, we recommend excluding model fits that use simple parameterization, as they performed significantly worse. However, it does not mean that more complex parameters are better, because too many complex parameters can lead to over‐fitting (Brun et al., 2020; Merow et al., 2014; Werkowska et al., 2017). Therefore, appropriate model complexity and sufficient sample size combined with linear DRTs for environmental variables are more conducive to the prediction performance of SDMs.

Algorithms of SDMs have been found to be one of the major drivers of uncertainty in predicting species potential distributions (Buisson et al., 2010; Garcia et al., 2012; Thuiller et al., 2019; Zhang & Wang, 2023). The SDMs algorithms selected in this study are all popular for predicting species distributions except ANN (Hao et al., 2019; Li & Wang, 2013). It is widely recognized that ANN is considered to be a “black box” model, as it does not provide a direct relationship between explanatory variables and response variables (Gobeyn et al., 2019; Kampichler et al., 2010). Even so, ANN has been shown to produce improved prediction accuracy for SDMs when combined with DRTs compared to other SDMs (Tables 2, S4 and S5). This may be because ANN is capable of reducing the dimensionality of data, as demonstrated by previous research (Hinton & Salakhutdinov, 2006).

In conclusion, our study demonstrates that DRTs can effectively improve the predictive performance of SDMs by reducing the dimensionality of environmental variables. Specifically, linear DRTs, especially PCA, were found to be more effective in improving model predictive performance under relatively complex model complexity or large sample sizes. Furthermore, previous research has suggested that DRTs work better as the dimension of variables increases (Reddy et al., 2020). Our study only utilized 45 environmental variables, which still have a certain gap with a large number of variables. Further studies could consider incorporating additional environmental variables to increase the dimensionality of the data and further investigate the impact of dimensionality reduction on model performance. In addition, we only considered plant species in this study, and we can extend it to animal species to study whether similar conclusions can be drawn in future studies.

## AUTHOR CONTRIBUTIONS

**Hao‐Tian Zhang:** Data curation (lead); formal analysis (lead); methodology (lead); writing – original draft (lead); writing – review and editing (equal). **Wen‐Yong Guo:** Writing – review and editing (equal). **Wen‐Ting Wang:** Conceptualization (lead); formal analysis (equal); project administration (lead); writing – original draft (equal); writing – review and editing (lead).

## FUNDING INFORMATION

This work was supported by the National Natural Science Foundation of China (no. 32260293), the Natural Science Foundation of Gansu Province (no. 21JR11RA023), the Scientific Research Project for Colleges and Universities of Gansu Province (no. 2022QB‐017), the Research Fund for Humanities and Social Sciences of the Ministry of Education (no. 20XJAZH006), and the Foundation Research Funds for the Central Universities (no. 31920220061; 31920220041).

## CONFLICT OF INTEREST STATEMENT

All authors disclose any potential sources of conflict of interest.

## Supporting information


Data S1.

## Data Availability

All codes required to reproduce our results are stored on GitHub (https://github.com/Hosky125/DRT). The cleaned occurrence records for the 23 real plant species and the generated occurrence records for the nine virtual species investigated in this study are: Dryad https://datadryad.org/stash/share/D78gyA4OEklVbDFKLuYjNy6wvZLrJybJvv8fcNwa89A.
