# Supplementary material for: The dimensionality reductions of environmental variables have a significant effect on the performance of species distribution models
Source: Ecol Evol. 2023 Nov 20;13(11):e10747. doi: 10.1002/ece3.10747 (PMC10659948; doi:10.1002/ece3.10747)
Supplement: Supplementary file 1 — Data S1. [file ECE3-13-e10747-s001.docx]

**Supporting Information**

to the paper " **The dimensionality reductions of environmental variables have a significant effect on the performance of species distribution models**"

**Table S1** Names, number of occurrence records, prevalence, and assigned sample size of species considered for 23 real plant species and nine virtual species. If the number of species occurrence records is less than 100, the species sample size is classified as small. If the number of species occurrence records is greater than 100 and less than 1000, the species sample size is classified as Middle. If the number of species occurrence records is greater than 1000, the species sample size is classified as big.

| Names | Abbreviation | Occurrence records | Prevalence | Sample size |
| --- | --- | --- | --- | --- |
| *Ageratum conyzoides* | *A. conyzoides* | 2242 | 0.098 | Big |
| *Alocasia macrorrhizos* | *A. macrorrhizos* | 460 | 0.057 | Middle |
| *Camellia japonica* | *C.  japonica* | 866 | 0.046 | Middle |
| *Ceratopteris thalictroides* | *C. thalictroides* | 984 | 0.068 | Middle |
| *Chamaecyparis obtuse* | *C. obtuse* | 643 | 0.026 | Middle |
| *Cinnamomum camphora* | *C. camphora* | 1546 | 0.018 | Big |
| *Erigeron canadensis* | *E. canadensis* | 3086 | 0.115 | Big |
| *Cypripedium flavum* | *C. flavum* | 59 | 0.029 | Small |
| *Selliguea dareiformis* | *S. dareiformis* | 51 | 0.062 | Small |
| *Herminium monorchis* | *H. monorchis* | 2087 | 0.049 | Big |
| *Impatiens balsamina* | *I. balsamina* | 2198 | 0.102 | Big |
| *Lycoris radiate* | *L. radiate* | 1103 | 0.045 | Big |
| *Meconopsis horridula* | *M. horridula* | 214 | 0.260 | Middle |
| *Meconopsis integrifolia* | *M. integrifolia* | 223 | 0.152 | Middle |
| *Meconopsis punicea* | *M. punicea* | 93 | 0.318 | Small |
| *Metasequoia glyptostroboides* | *M. glyptostroboides* | 642 | 0.021 | Middle |
| *Oxalis corniculata* | *O. corniculata* | 3531 | 0.093 | Big |
| *Passiflora siamica* | *P. siamica* | 59 | 0.158 | Small |
| *Pinellia ternate* | *P. ternate* | 606 | 0.054 | Middle |
| *Pinus armandii* | *P. armandii* | 224 | 0.072 | Middle |
| *Cerasus serrulata* | *C. serrulata* | 1327 | 0.042 | Big |
| *Sphagnum squarrosum* | *S. squarrosum* | 7007 | 0.063 | Big |
| *Taiwania cryptomerioides* | *T. cryptomerioides* | 81 | 0.008 | Small |
| Virtual Species1 |  | 93 | 0.318 | Small |
| Virtual Species2 |  | 3086 | 0.115 | Big |
| Virtual Species3 |  | 984 | 0.068 | Middle |
| Virtual Species4 |  | 2087 | 0.049 | Big |
| Virtual Species5 |  | 1103 | 0.045 | Big |
| Virtual Species6 |  | 81 | 0.008 | Small |
| Virtual Species7 |  | 643 | 0.026 | Middle |
| Virtual Species8 |  | 224 | 0.072 | Middle |
| Virtual Species9 |  | 59 | 0.158 | Small |

**Table S2** The characteristics of the 23 real plants included habitat, range size, climatic zone, altitude, plant type, habit and whether it was a vascular plant, as well as the source and citation of the species occurrence records.

| Abbreviation | Habitats | Range size | Climate zone | Altitude | Plant type | Habit | Vascular plant | Sources and citation |
| --- | --- | --- | --- | --- | --- | --- | --- | --- |
| *A. conyzoides* | Terrestrial | Global | Tropical | >3500 | Annual herb | Thermophilic | Y | https://doi.org/10.15468/dl.m6nzsr |
| *A. macrorrhizos* | Terrestrial | Global | Tropical | <1000 | Perennial herb | Ombrophyte and hygrophilo | Y | https://doi.org/10.15468/dl.a2d9n5 |
| *C. japonica* | Terrestrial | Global | Tropical and subtropical | <800 | Shrub | Thermophilic and hygrophilo | Y | https://doi.org/10.15468/dl.aefvfh |
| *C. thalictroides* | Aquatic | Global | Tropical and subtropical | <1000 | Aquatic plant | Thermophilic and hygrophilo | Y | https://doi.org/10.15468/dl.9mnyp4 |
| *C. obtuse* | Terrestrial | Continental | Subtropical | 1300-2800 | Evergreen | Ombrophyte and hygrophilo | Y | https://doi.org/10.15468/dl.fqqutr |
| *C. camphora* | Terrestrial | Global | Subtropical | <1500 | Evergreen | Thermophilic and hygrophilo | Y | https://doi.org/10.15468/dl.yks4rm |
| *E. canadensis* | Terrestrial | Global | Temperate | 30-200 | Perennial herb | Xerophilous | Y | https://doi.org/10.15468/dl.pnfne8 |
| *C. flavum* | Terrestrial | Local | Temperate and subtropical | 1800-3450 | Perennial herb | Hygrophilo | Y | https://doi.org/10.15468/dl.jtdpsa |
| *S. dareiformis* | Terrestrial | Local | Tropical and subtropical | 1300-2700 | Filicophytin | Thermophilic and hygrophilo | Y | https://doi.org/10.15468/dl.xddrgu |
| *H. monorchis* | Terrestrial | Continental | Temperate and subtropical | 600-4500 | Perennial herb | Hygrophilo | Y | https://doi.org/10.15468/dl.jy6u4g |
| *I. balsamina* | Terrestrial | Global | Tropical and subtropical | <1000 | Annual herb | Photophilous and thermophyte | Y | https://doi.org/10.15468/dl.tsh7kk |
| *L. radiate* | Terrestrial | Continental | Subtropical and temperate | <1200 | Perennial herb | Thermophilic and hygrophilo | Y | https://doi.org/10.15468/dl.yb3wsm |
| *M. horridula* | Terrestrial | Local | Plateau climate | 3600-5100 | Annual herb | Psychrophi | Y | https://doi.org/10.15468/dl.f6r964 |
| *M. integrifolia* | Terrestrial | Local | Plateau climate | 2700-5100 | Annual, biennial or perennial herbs | Psychrophi and hygrophilo | Y | https://doi.org/10.15468/dl.w8ss39 |
| *M. punicea* | Terrestrial | Local | Plateau climate | 2800-4300 | Perennial herb | Psychrophi and hygrophilo | Y | https://doi.org/10.15468/dl.zbmv2c |
| *M. glyptostroboides* | Terrestrial | Continental | Temperate and subtropical | <1500 | Deciduous tree | Hygrophilo | Y | https://doi.org/10.15468/dl.x3jnmf |
| *O. corniculata* | Terrestrial | Global | Tropical and subtropical | 10-350 | Perennial herb | Thermophilic and hygrophilo | Y | https://doi.org/10.15468/dl.nwd57h |
| *P. siamica* | Terrestrial | Local | Tropical and subtropical | 540-1600 | Liane | Thermophilic and hygrophilo | Y | https://doi.org/10.15468/dl.byrbaj |
| *P. ternate* | Terrestrial | Continental | Temperate and subtropical | <2500 | Perennial herb | Thermophilic and hygrophilo | Y | https://doi.org/10.15468/dl.pxchzg |
| *P. armandii* | Terrestrial | Local | Temperate | 1500-3000 | Evergreen | Thermophilic and hygrophilo | Y | https://doi.org/10.15468/dl.p7t386 |
| *C. serrulata* | Terrestrial | Continental | Temperate and subtropical | 400-800 | Deciduous tree | Thermophilic and hygrophilo | Y | https://doi.org/10.15468/dl.92csxe |
| *S. squarrosum* | Wetland | Global | Frigid and temperate | <1000 | Bryophyta | Hygrophilo | N | https://doi.org/10.15468/dl.axwe83 |
| *T. cryptomerioides* | Terrestrial | Local | Tropical monsoon | 1500-2500 | Evergreen | Thermophilic and hygrophilo | Y | https://doi.org/10.15468/dl.ahye78 |

**Table S3** The environmental variables for our analysis were the 19 bioclimatic variables of current climate (representative of 1970–2000) with an original resolution of 2.5 arc-min and elevation data at a raw resolution of 30 arc-sec taken from the WorldClim database version 2.1 (http://www.world clim.org/), and terrain ruggedness (maximum elevation difference in each 5 × 5 km^2^ cell), topographic position index (difference between the elevation of a cell and the mean value of its 24 surrounding cells; each cell is 1 × 1 km^2^), mean slope, maximum difference of slope in each 5 × 5 km^2^ cell, and aspect, derived from the elevation data, 18 soil variables were obtained from the Harmonized World Soil Database (HWSD) with a spatial resolution of 30 arc-sec, and data representing ecological indicators, including potential evapotranspiration (PET) and the aridity index (AI), were acquired at a resolution of 30 arc-sec from Version 3 of the Global Aridity Index and Potential Evapotranspiration Database. Abbreviations, full names and sources of 45 environmental variables are listed.

| Abbreviation | Full names | Sources |
| --- | --- | --- |
| BIO1 | Annual Mean Temperature | WorldClim database  (http://www.world clim.org/) |
| BIO2 | Mean Diurnal Range (Mean of monthly (max temp – min temp)) |  |
| BIO3 | Isothermality (BIO2/BIO7) |  |
| BIO4 | Temperature Seasonality (standard deviation×100) |  |
| BIO5 | Max Temperature of Warmest Month |  |
| BIO6 | Min Temperature of Coldest Month |  |
| BIO7 | Temperature Annual Range (BIO5 – BIO6) |  |
| BIO8 | Mean Temperature of Wettest Quarter |  |
| BIO9 | Mean Temperature of Driest Quarter |  |
| BIO10 | Mean Temperature of Warmest Quarter |  |
| BIO11 | Mean Temperature of Coldest Quarter |  |
| BIO12 | Annual Precipitation |  |
| BIO13 | Precipitation of Wettest Month |  |
| BIO14 | Precipitation of Driest Month |  |
| BIO15 | Precipitation Seasonality (Coefficient of Variation) |  |
| BIO16 | Precipitation of Wettest Quarter |  |
| BIO17 | Precipitation of Driest Quarter |  |
| BIO18 | Precipitation of Warmest Quarter |  |
| BIO19 | Precipitation of Coldest Quarter |  |
| T1 | Topsoil USDA Texture Classification USDA (T_USDA_TEX_CLASS) | Harmonized Word Soil Database |
| T2 | Topsoil Texture (T_TEXTURE) |  |
| T3 | Topsoil TEB (T_TEB) |  |
| T4 | Topsoil Silt Fraction (T_SILT) |  |
| T5 | Topsoil Sand Fraction (T_SAND) |  |
| T6 | Topsoil pH (H2O) (T_PH_H2O) |  |
| T7 | Topsoil Organic Carbon (T_OC) |  |
| T8 | Topsoil Gravel Content (T_GRAVEL) |  |
| T9 | Topsoil Sodicity (ESP) (T_ESP) |  |
| T10 | Topsoil Salinity (Elco) (T_ECE) |  |
| T11 | Topsoil Clay Fraction (T_CLAY) |  |
| T12 | Topsoil CEC (soil) (T_CEC_SOIL) |  |
| T13 | Topsoil CEC (clay) (T_CEC_CLAY) |  |
| T14 | Topsoil Gypsum (T_CASO4) |  |
| T15 | Topsoil Calcium Carbonate (T_CACO3) |  |
| T16 | Topsoil Base Saturation (T_BS) |  |
| T17 | Topsoil Reference Bulk Density (T_REF_BULK_DENSITY) |  |
| T18 | Topsoil Bulk Density (T_BULK_DENSITY) |  |
| AI | Aridity Index | Global Aridity Index and Potential Evapotranspiration Database (https://www.plantplus.cn/cn/dataset) |
| PET | Potential evapotranspiration |  |
| ELE | Elevation | Elevation (ELE) is derived from WordClim Database (http://www.worldclim.org/), and several other variables are computed by ELE. |
| MA | Mean aspect |  |
| MS | Mean slope |  |
| SR | Slope Roughness is the difference between the maximum slope and the minimum slope value of a cell and its 24 surrounding cells |  |
| ER | Roughness is the difference between the maximum elevation and the minimum elevation value of a cell and its 24 surrounding cells |  |
| TPI | Topographic Position Index (TPI) is the difference between the elevation value of a cell and the mean elevation value of its 24 surrounding cells. |  |

**Table S4** The predictive performance (as measured by AUC) of all models at different Pearson's correlation coefficient (PCC) criteria (PCC less than 0.70, PCC less than 0.75, and PCC less than 0.80) for each species, and the numbers in the table represent the median of a set of AUC values.

| Species | Environmental variables selected by PCC | | |
| --- | --- | --- | --- |
|  | <0.70 | <0.75 | <0.80 |
| *A. conyzoides* | 0.9238704 | 0.9233552 | **0.9241511** |
| *A. macrorrhizos* | 0.9081546 | **0.908197** | 0.9073069 |
| *C. japonica* | 0.9618421 | 0.9626664 | **0.9627554** |
| *C. thalictroides* | 0.9153098 | 0.9174397 | **0.9183748** |
| *C. obtuse* | 0.9856322 | 0.9860804 | **0.9863286** |
| *C. camphora* | 0.9690025 | **0.9717616** | 0.9714769 |
| *E. canadensis* | 0.9102625 | 0.9127564 | **0.9132458** |
| *C. flavum* | 0.9431818 | **0.945592** | 0.9431818 |
| *S. dareiformis* | 0.7896552 | 0.7896552 | **0.8000000** |
| *H. monorchis* | 0.9487168 | 0.9488388 | **0.9488434** |
| *I. balsamina* | **0.9153727** | 0.9106901 | 0.914189 |
| *L. radiate* | 0.9846703 | 0.9857425 | **0.9860639** |
| *M. horridula* | 0.8115861 | 0.8146794 | **0.8161792** |
| *M. integrifolia* | 0.8012636 | 0.8007547 | **0.8063094** |
| *M. punicea* | 0.7718254 | **0.7946429** | 0.7936508 |
| *M. glyptostroboides* | **0.9726308** | 0.9721378 | 0.9711918 |
| *O. corniculata* | 0.9082893 | 0.909436 | **0.9096335** |
| *P. siamica* | 0.8241379 | 0.8206897 | **0.8275862** |
| *P. ternate* | 0.9853016 | 0.9849691 | **0.9853505** |
| *P. armandii* | 0.8972129 | 0.896414 | **0.8973016** |
| *C. serrulata* | 0.9796553 | 0.9793693 | **0.9806149** |
| *S. squarrosum* | 0.966467 | 0.9666535 | **0.9666957** |
| *T. cryptomerioides* | 0.8936335 | **0.9037267** | 0.8994565 |
| Virtual Species1 | 0.8631546 | **0.863197** | 0.8623069 |
| Virtual Species2 | 0.8981818 | **0.900592** | 0.8981818 |
| Virtual Species3 | 0.8346552 | 0.8346552 | **0.8458765** |
| Virtual Species4 | 0.7665861 | 0.7696794 | **0.7711792** |
| Virtual Species5 | 0.7562636 | 0.7557547 | **0.7613094** |
| Virtual Species6 | 0.7218254 | **0.7446429** | 0.7436508 |
| Virtual Species7 | 0.9353016 | 0.9349691 | **0.9353505** |
| Virtual Species8 | **0.8653727** | 0.8606901 | 0.864189 |
| Virtual Species9 | **0.9226308** | 0.9221378 | 0.9211918 |

**Table S5** For each species, we retained for subsequent comparison analyses environmental variables with different Pearson's correlation coefficient (PCC) criteria. The PCC criteria for each species are according to Table S4.

| Species | Environmental variables selected by PCC |
| --- | --- |
| *A. conyzoides* | BIO2, BIO7, BIO10, BIO11, BIO15, BIO16, BIO17, BIO18, BIO19, T3, T7, T8, T9, T10, T13, T14, T15, T16, T17, T18, ELE, TPI, SR, MA, ETO, AI |
| *A. macrorrhizos* | BIO2, BIO5, BIO7, BIO11, BIO16, BIO17, BIO18, BIO19, T3, T8, T13, T14, T15, T16, T17, T18, ELE, TPI, SR, MA, ETO, AI |
| *C. japonica* | BIO2, BIO5, BIO8, BIO11, BIO13, BIO15, BIO17, BIO18, BIO19, T3, T8, T9, T10, T13, T14, T15, T16, T17, T18, ELE, TPI, SR, MA, ETO, AI |
| *C. thalictroides* | BIO2, BIO10, BIO11, BIO15, BIO16, BIO17, BIO18, BIO19, T3, T8, T13, T14, T15, T16, T17, T18, ELE, TPI, SR, MA, ETO, AI |
| *C. obtuse* | BIO3, BIO5, BIO7, BIO8, BIO11, BIO15, BIO19, T3, T6, T8, T13, T14, T15, T17, T18, TPI, SR, MA, ETO, AI |
| *C. camphora* | BIO2, BIO7, BIO8, BIO10, BIO11, BIO15, BIO19, T3, T8, T13, T14, T15, T16, T17, T18, ELE, TPI, SR, MA, ETO, AI |
| *C. flavum* | BIO2, BIO7, BIO8, BIO10, BIO11, BIO15, BIO16, BIO17, BIO18, BIO19, T3, T8, T9, T13, T14, T15, T16, T17, T18, ELE, TPI, SR, MA, ETO, AI |
| *E. canadensis* | BIO2, BIO7, BIO11, BIO18, BIO19, T3, T4, T8, T9, T13, T14, T15, T16, T17, T18, ELE, TPI, SR, MA, ETO, AI |
| *S. dareiformis* | BIO2, BIO4, BIO7, BIO15, BIO19, T8, T9, T13, T14, T15, T17, T18, ELE, TPI, SR, MA, ETO, AI |
| *H. monorchis* | BIO3, BIO8, BIO10, BIO11, BIO15, BIO18, T3, T8, T10, T13, T14, T15, T16, T17, T18, ELE, TPI, SR, MA, ETO, AI |
| *I. balsamina* | BIO2, BIO6, BIO11, BIO16, BIO17, BIO18, BIO19, T3, T8, T13, T14, T15, T16, T17, T18, ELE, TPI, SR, MA, ETO, AI |
| *L. radiate* | BIO3, BIO7, BIO8, BIO9, BIO10, BIO11, BIO17, BIO19, T3, T8, T13, T15, T16, T17, T18, ELE, TPI, SR, MA, ETO, AI |
| *M. horridula* | BIO2, BIO5, BIO7, BIO8, BIO9, BIO11, BIO17, BIO19, T3, T4, T8, T9, T13, T14, T15, T16, T17, T18, ELE, TPI, SR, MA, ETO, AI |
| *M. integrifolia* | BIO2, BIO3, BIO7, BIO11, BIO13, BIO15, BIO19, T4, T8, T9, T13, T14, T15, T16, T17, T18, ELE, TPI, SR, MA, ETO, AI |
| *M. punicea* | BIO2, BIO3, BIO12, BIO15, BIO19, T5, T8, T9, T13, T14, T15, T16, T17, T18, ELE, TPI, SR, MA, ETO, AI |
| *M. glyptostroboides* | BIO3, BIO8, BIO10, BIO11, BIO15, BIO18, BIO19, T8, T9, T10, T13, T14, T15, T16, T17, T18, ELE, TPI, SR, MA, ETO, AI |
| *O. corniculata* | BIO2, BIO7, BIO8, BIO9, BIO10, BIO11, BIO15, BIO17, BIO18, BIO19, T3, T8, T10, T13, T14, T15, T16, T17, T18, ELE, TPI, SR, MA, ETO, AI |
| *P. siamica* | BIO2, BIO4, BIO7, BIO9, BIO15, BIO19, T8, T13, T14, T15, T16, T17, T18, ELE, TPI, SR, MA, ETO, AI |
| *P. ternate* | BIO2, BIO3, BIO8, BIO10, BIO11, BIO15, BIO18, BIO19, T3, T8, T9, T10, T13, T14, T15, T16, T17, T18, ELE, TPI, SR, MA, ETO, AI |
| *P. armandii* | BIO2, BIO3, BIO7, BIO11, BIO15, T3, T4, T7, T8, T9, T10, T13, T14, T15, T16, T17, T18, ELE, TPI, SR, MA, ETO, AI |
| *C. serrulata* | BIO1, BIO2, BIO3, BIO7, BIO10, BIO11, BIO15, BIO18, BIO19, T3, T8, T9, T10, T13, T14, T15, T16, T17, T18, ELE, TPI, SR, MA, ETO, AI |
| *S. squarrosum* | BIO2, BIO8, BIO10, BIO11, BIO14, BIO15, BIO18, BIO19, T3, T8, T10, T13, T14, T15, T16, T17, T18, ELE, TPI, SR, MA, ETO, AI |
| *T. cryptomerioides* | BIO3, BIO11, BIO12, BIO18, BIO19, T8, T9, T10, T13, T14, T15, T16, T17, T18, ELE, TPI, SR, MA, ETO, AI |
| Virtual Species1 | BIO2, BIO7, BIO8, BIO10, BIO11, BIO15, BIO17, BIO18, BIO19, T3, T8, T10, T13, T14, T15, T16, T17, T18, ELE, TPI, SR, MA, ETO, AI |
| Virtual Species2 | BIO3, BIO10, BIO11, BIO17, BIO19, T3, T8, T13, T14, T15, T17, T18, ELE, TPI, SR, MA, ETO, AI |
| Virtual Species3 | BIO2, BIO7, BIO11, BIO18, BIO19, T3, T8, T9, T13, T14, T15, T16, T17, T18, ELE, TPI, SR, MA, ETO, AI |
| Virtual Species4 | BIO2, BIO3, BIO12, BIO15, BIO19, T3, T4, T8, T9, T13, T14, T15, T16, T17, T18, ELE, TPI, SR, MA, ETO, AI |
| Virtual Species5 | BIO2, BIO3, BIO7, BIO8, BIO9, BIO10, BIO11, BIO15, BIO18, BIO19, T3, T8, T9, T10, T13, T14, T15, T16, T17, T18, ELE, TPI, SR, MA, ETO, AI |
| Virtual Species6 | BIO4, BIO7, BIO15, BIO19, T8, T13, T14, T15, T17, T18, ELE, TPI, SR, MA, ETO, AI |
| Virtual Species7 | BIO2, BIO3, BIO11, BIO12, BIO15, T3, T7, T8, T9, T10, T13, T14, T15, T16, T17, T18, ELE, TPI, SR, MA, ETO, AI |
| Virtual Species8 | BIO2, BIO7, BIO11, BIO18, BIO19, T3, T8, T9, T10, T13, T14, T15, T16, T17, T18, ELE, TPI, SR, MA, ETO, AI |
| Virtual Species9 | BIO2, BIO9, BIO10, BIO11, BIO13, BIO16, BIO19, T3, T8, T9, T10, T13, T14, T15, T16, T17, T18, ELE, TPI, SR, MA, ETO, AI |

**Table S6** The percentage improvement in species distribution models (SDMs, i.e., GLM, GBM, FDA, RF, and ANN) predictive performance (measured by KAPPA) using four dimensionality reduction techniques (DRTs, i.e., PCA, ICA, UMAP, and KPCA) compared with using Pearson's correlation coefficient (PCC).

|  | GLM | GBM | FDA | RF | ANN |
| --- | --- | --- | --- | --- | --- |
| PCA-PCC | 4.25% | 4.08% | 5.28% | 4.37% | 10.55% |
| ICA-PCC | 4.89% | 2.53% | 4.43% | 4.24 % | 13.74% |
| UMAP-PCC | 3.54% | 3.28% | 3.13% | 3.29% | 14.23% |
| KPCA-PCC | –11.79% | –12.56% | –11.62% | –10.67% | –8.97% |

**Table S7** The percentage improvement in species distribution models (SDMs, i.e., GLM, GBM, FDA, RF, and ANN) predictive performance (measured by TSS) using four dimensionality reduction techniques (DRTs, i.e., PCA, ICA, UMAP, and KPCA) compared with using Pearson's correlation coefficient (PCC).

|  | GLM | GBM | FDA | RF | ANN |
| --- | --- | --- | --- | --- | --- |
| PCA-PCC | 4.63% | 4.44% | 5.91% | 4.51% | 11.58% |
| ICA-PCC | 3.27% | 4.17% | 4.29% | 4.78% | 13.03% |
| UMAP-PCC | 4.49% | 3.43% | 3.19% | 4.27% | 11.38% |
| KPCA-PCC | –12.64% | –13.24% | –12.15% | –10.39% | –9.53% |

**Table S8** The percentage improvement in SDMs predictive performance (measured by KAPPA) using different dimensionality reduction techniques (i.e., PCA, ICA, UMAP, and KPCA) and Pearson's correlation coefficient (PCC) under different model complexity (i.e., simple, intermediate, and complex) or sample size (i.e., small, middle, and big).

|  | Model complexity | | | Sample size | | |
| --- | --- | --- | --- | --- | --- | --- |
|  | Simple | Intermediate | Complex | Small | Middle | Big |
| PCA-PCC | 1.66% | 2.34% | 2.79% | 1.34% | 2.22% | 2.03% |
| ICA-PCC | 1.03% | 2.31% | 2.58% | 0.91% | 1.37% | 1.82% |
| UMAP-PCC | 1.14% | 2.07% | 2.36% | 1.21% | 1.59% | 1.32% |
| KPCA-PCC | –13.26% | –13.03% | –13.27% | –11.64% | –10.32% | –9.81% |

**Table S9** The percentage improvement in SDMs predictive performance (measured by TSS) using different dimensionality reduction techniques (i.e., PCA, ICA, UMAP, and KPCA) and Pearson's correlation coefficient (PCC) under different model complexity (i.e., simple, intermediate, and complex) or sample size (i.e., small, middle, and big).

|  | Model complexity | | | Sample size | | |
| --- | --- | --- | --- | --- | --- | --- |
|  | Simple | Intermediate | Complex | Small | Middle | Big |
| PCA-PCC | 2.55% | 2.76% | 2.85% | 1.83% | 2.47% | 2.36% |
| ICA-PCC | 1.34% | 2.19% | 2.27% | 1.11% | 1.24% | 1.57% |
| UMAP-PCC | 1.78% | 1.53% | 1.92% | 1.73% | 2.26% | 1.45% |
| KPCA-PCC | –10.34% | –11.64% | –10.31% | –10.29% | –11.32% | –10.27% |

*
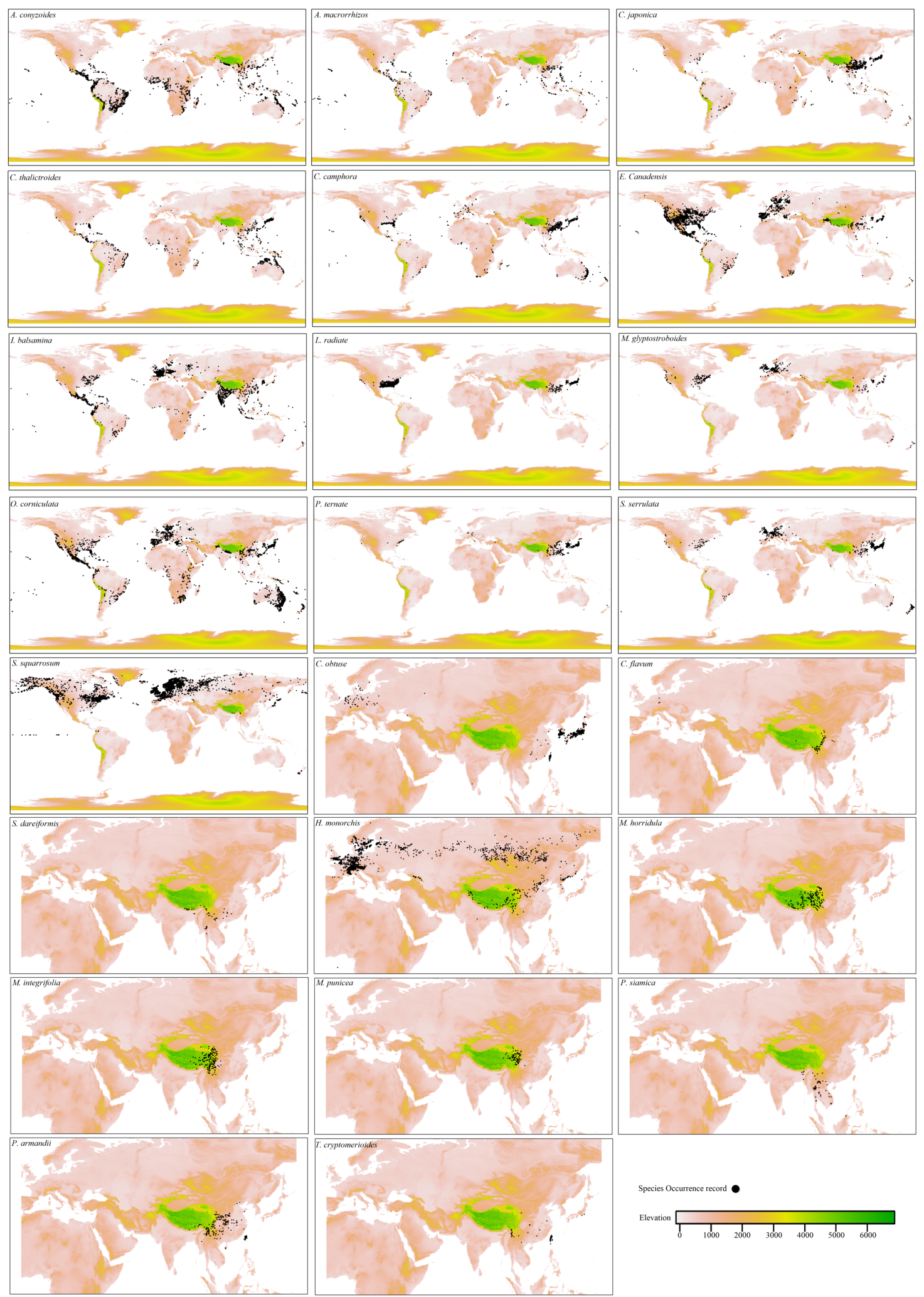
*

**Figure S1** Occurrence data for 23 real species used in this study. The number of records per species can be found in Table S1.

**
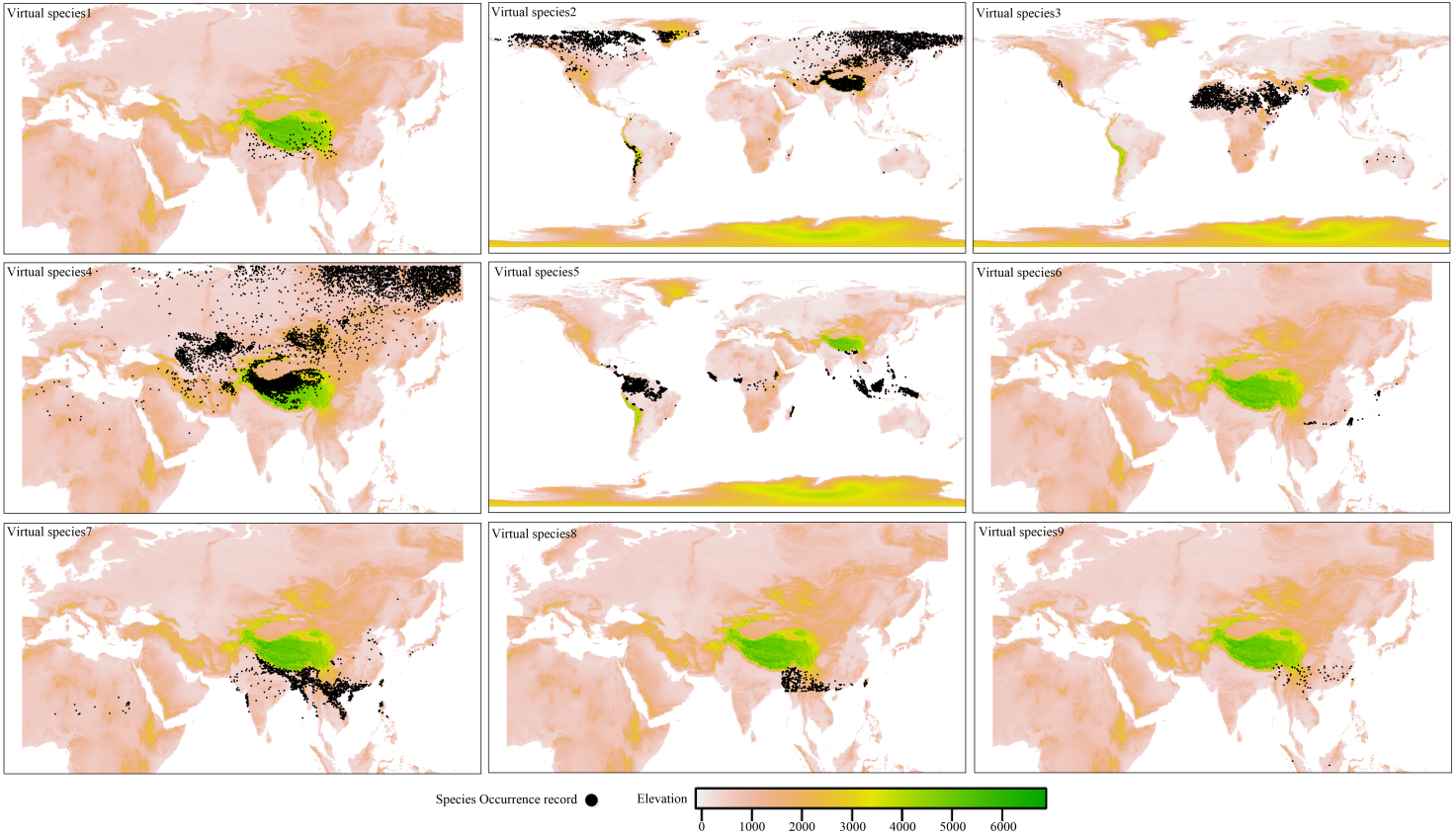
**

**Figure S2** Occurrence data for nine virtual species used in this study. The number of records per species can be found in Table S1.

**
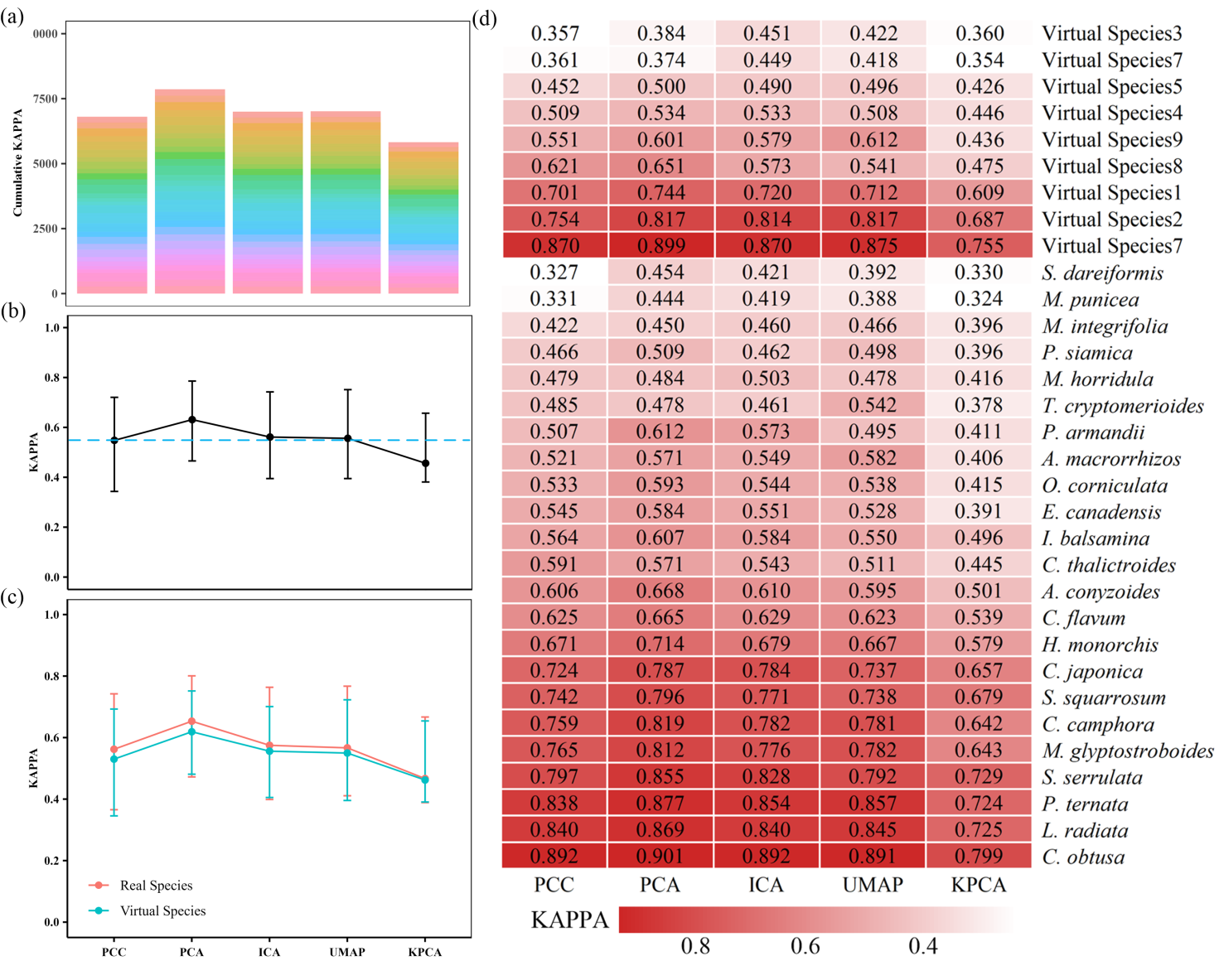
**

**Figure S3** Statistical results of the predictive performance (measured by KAPPA) of five species distribution models (SDMs, i.e., GLM, GBM, RF, FDA, and ANN) constructed after the environmental variables were treated with the four dimensionality reduction techniques (DRTs, i.e., PCA, ICA, UMAP, and KPCA) and Pearson's correlation coefficient (PCC). (a) The cumulative KAPPA value for all SDMs predicting 32 species distributions under each DRT and PCC. (b) The median of KAPPA values for all SDMs predicting 32 species distributions under each DRT and PCC. (c) The median of KAPPA values for all SDMs predicting species distribution of real and virtual species under each DRT and PCC. (d) The median of KAPPA values for all SDMs predicting species distribution of each species under each DRT and PCC, the row and the column represents the median of the prediction results for different species and under five DRTs, respectively. See Table S1 for specific species names.

**
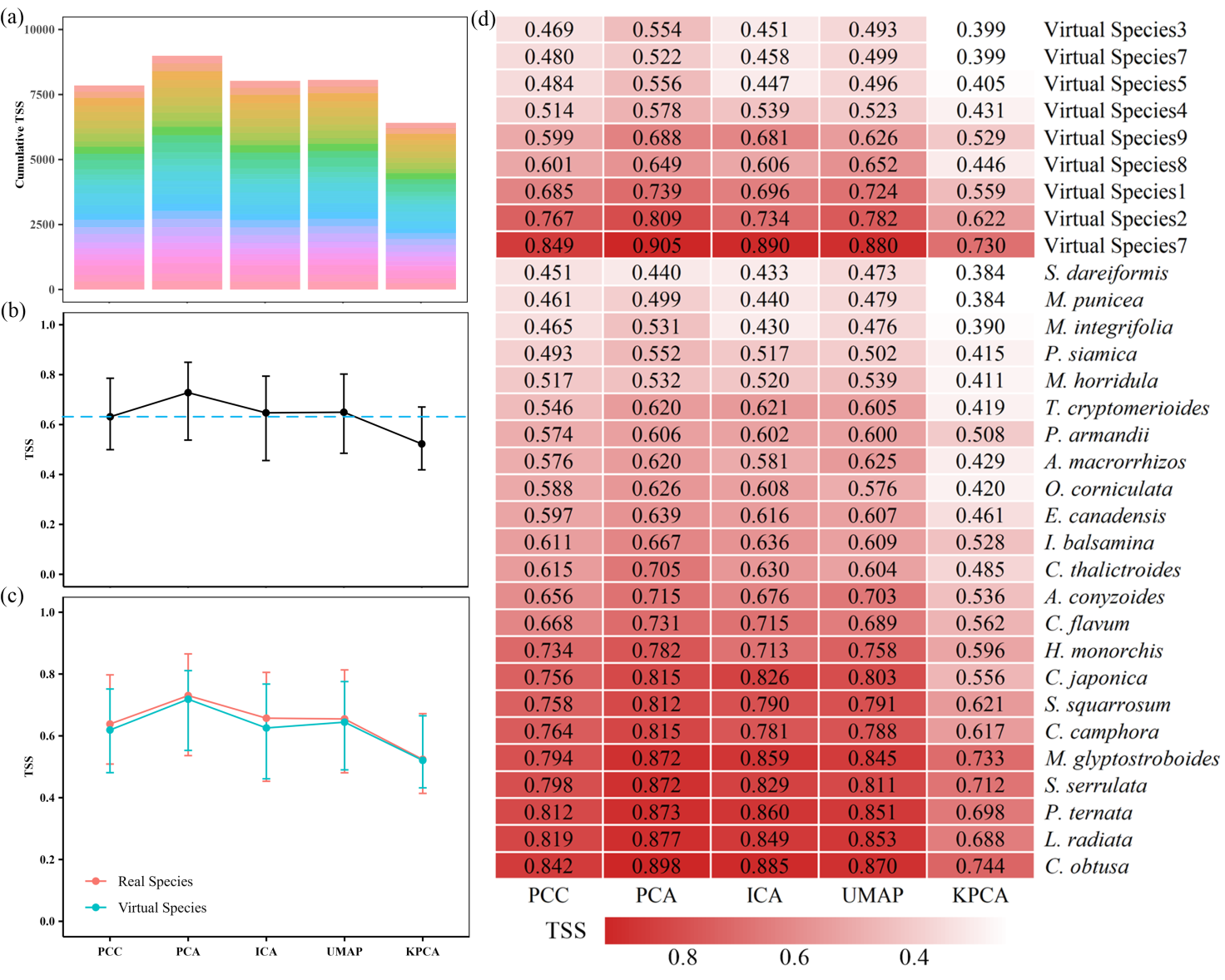
**

**Figure S4** Statistical results of the predictive performance (measured by TSS) of five species distribution models (SDMs, i.e., GLM, GBM, RF, FDA, and ANN) constructed after the environmental variables were treated with the four dimensionality reduction techniques (DRTs, i.e., PCA, ICA, UMAP, and KPCA) and Pearson's correlation coefficient (PCC). (a) The cumulative TSS value for all SDMs predicting 32 species distributions under each DRT and PCC. (b) The median of TSS values for all SDMs predicting 32 species distributions under each DRT and PCC. (c) The median of TSS values for all SDMs predicting species distribution of real and virtual species under each DRT and PCC. (d) The median of TSS values for all SDMs predicting species distribution of each species under each DRT and PCC, the row and the column represents the median of the prediction results for different species and under five DRTs, respectively. See Table S1 for specific species names.

**
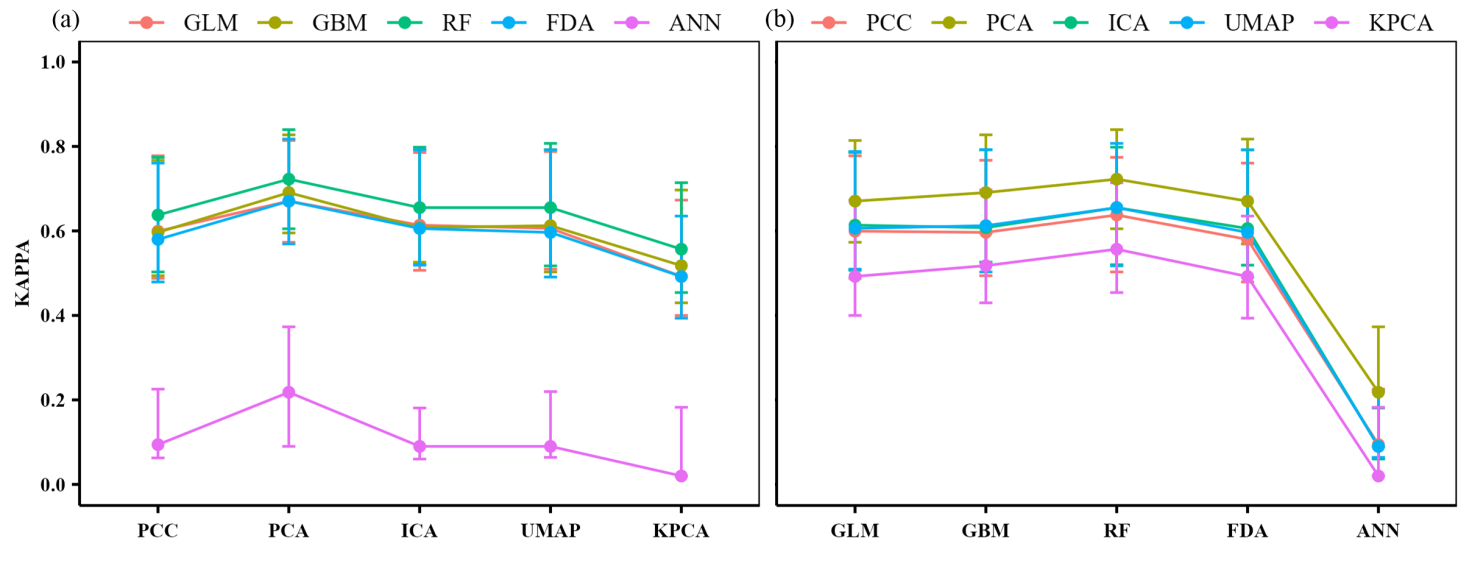
**

**Figure S5** Statistical results of predictive performance (measured by KAPPA) of five species distribution models (SDMs, i.e., GLM, GBM, RF, FDA, and ANN) constructed after the environmental variables were treated with the four dimensionality reduction techniques (DRTs, i.e., PCA, ICA, UMAP, and KPCA) and Pearson's correlation coefficient (PCC). (a) The median of KAPPA values for different SDMs predicting 32 species distributions under each DRT and PCC. (b) The median of KAPPA values for each SDM predicting 32 species distributions under different DRTs and PCC.

**
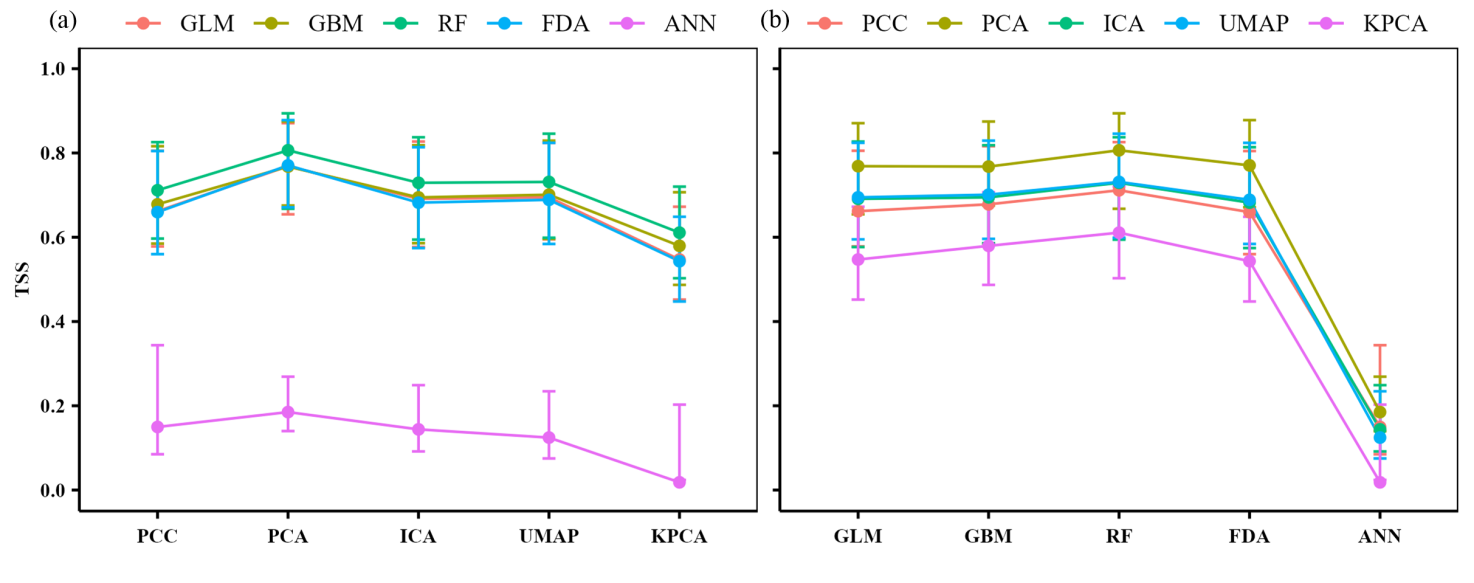
**

**Figure S6** Statistical results of predictive performance (measured by TSS) of five species distribution models (SDMs, i.e., GLM, GBM, RF, FDA, and ANN) constructed after the environmental variables were treated with the four dimensionality reduction techniques (DRTs, i.e., PCA, ICA, UMAP, and KPCA) and Pearson's correlation coefficient (PCC). (a) The median of TSS values for different SDMs predicting 32 species distributions under each DRT and PCC. (b) The median of TSS values for each SDM predicting 32 species distributions under different DRTs and PCC.


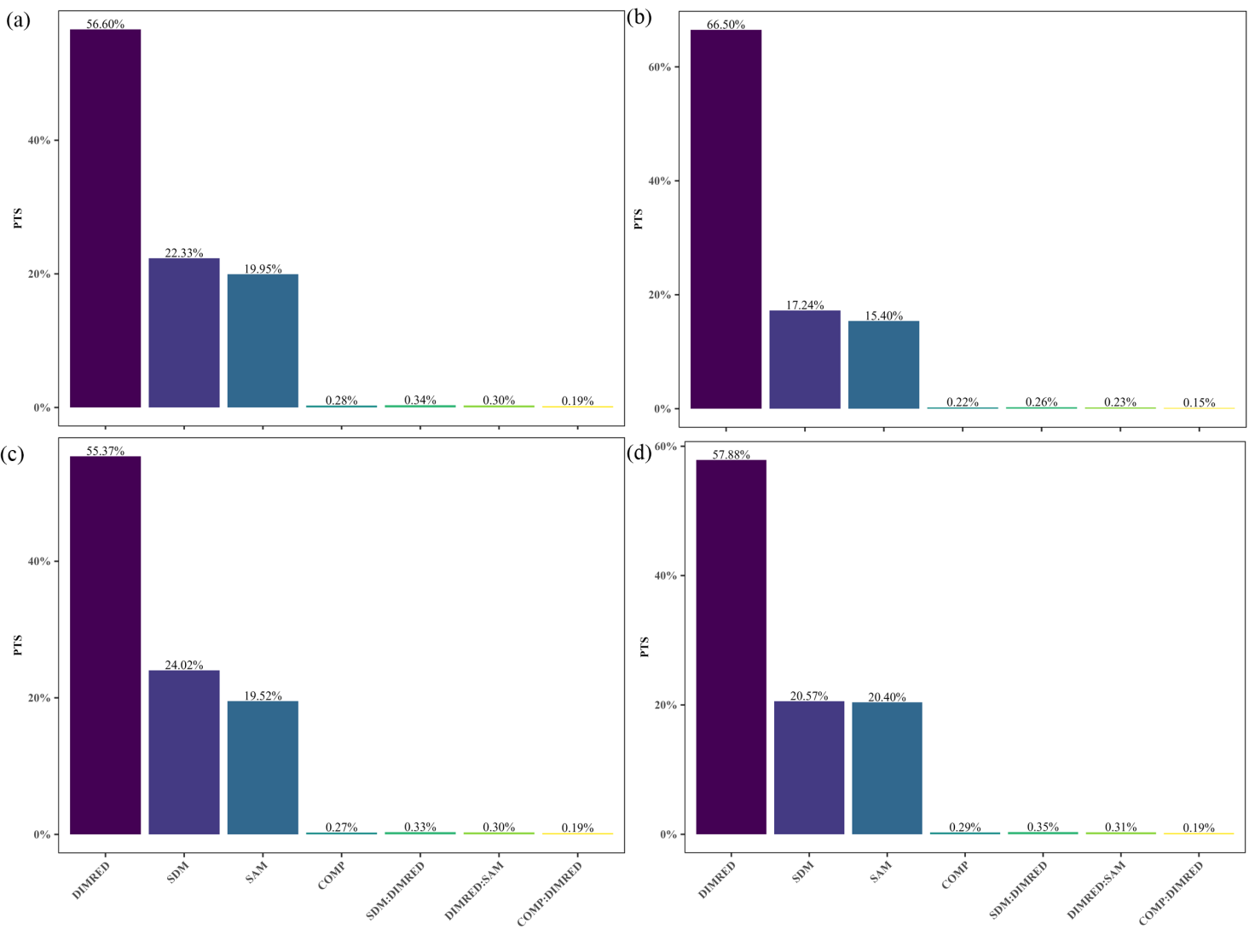


**Figure S7** Relative contributions of different influencing factor on predictive performance assessed by MANOVA, and the evaluation metric is the area under the receiver operating characteristic curve (KAPPA). species distribution model (SDM) and dimensionality reduction technique (DRT) among the influencing factors, considers four scenarios: (a) including all SDMs (i.e., GLM, GBM, RF, FDA, ANN) and DRTs (i.e., PCA, ICA, UMAP, KPCA) considered in this study, (b) ANN in SDMs is removed, (c) KPCA in DRTs is removed, and (d) ANN in SDMs and KPCA in DRTs are both removed. Bars represent the proportion of total sums of squares (PTS). The bars labeled ‘COMP’, ‘DIMRED’, ‘SDM’, and ‘SAM’ respectively represent model complexity, dimensionality reduction, the SDM algorithm, and sample size. The bars labeled ‘COMP:DIMRED’, ‘SAM:DIMRED’, and ‘SDM:DIMRED’ respectively represent the interaction between model complexity and dimensionality reduction, the interaction between sample size and dimensionality reduction, as well as the interaction between the SDM algorithm and dimensionality reduction.


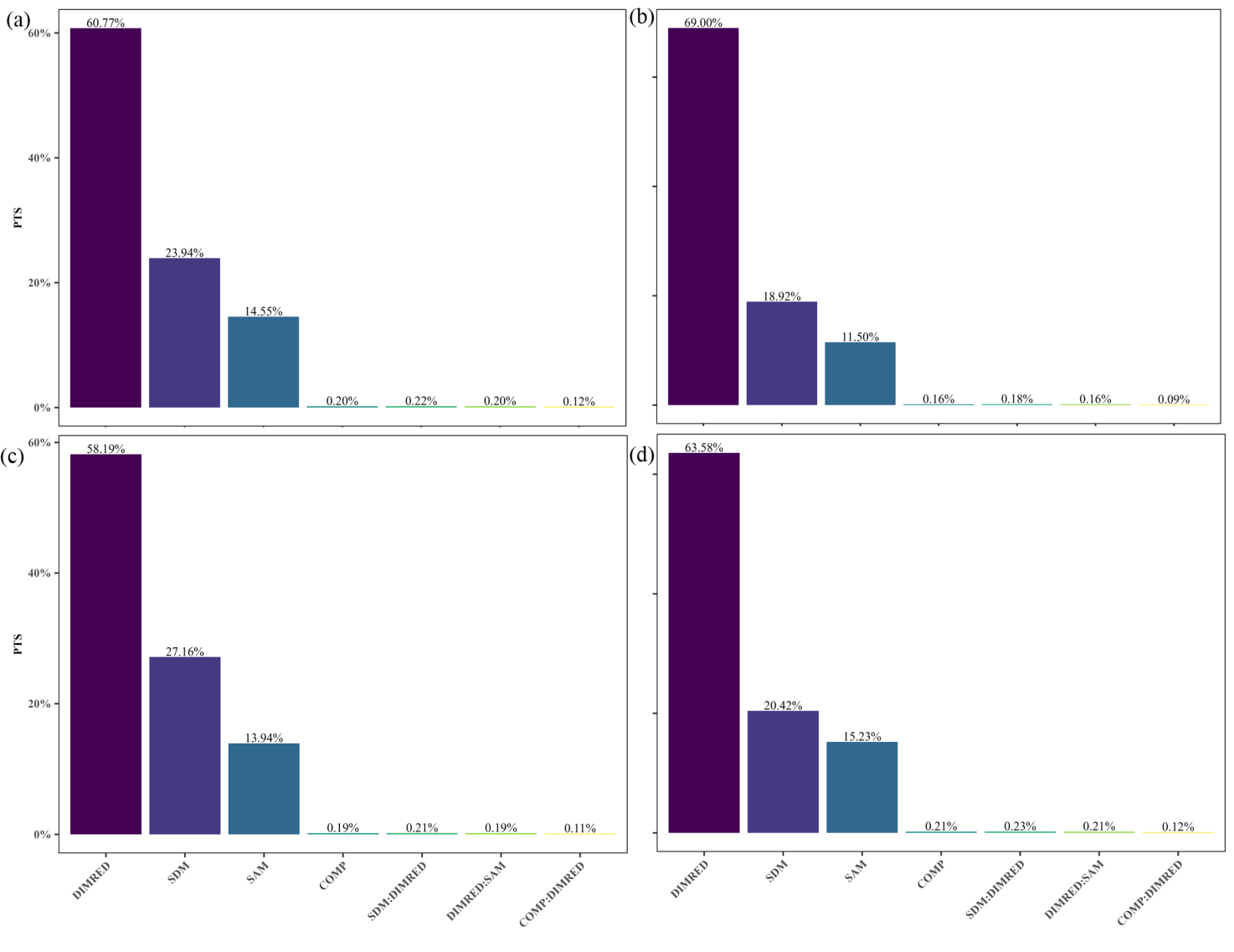


**Figure S8** Relative contributions of different influencing factor on predictive performance assessed by MANOVA, and the evaluation metric is the area under the receiver operating characteristic curve (TSS). species distribution model (SDM) and dimensionality reduction technique (DRT) among the influencing factors, considers four scenarios: (a) including all SDMs (i.e., GLM, GBM, RF, FDA, ANN) and DRTs (i.e., PCA, ICA, UMAP, KPCA) considered in this study, (b) ANN in SDMs is removed, (c) KPCA in DRTs is removed, and (d) ANN in SDMs and KPCA in DRTs are both removed. Bars represent the proportion of total sums of squares (PTS). The bars labeled ‘COMP’, ‘DIMRED’, ‘SDM’, and ‘SAM’ respectively represent model complexity, dimensionality reduction, the SDM algorithm, and sample size. The bars labeled ‘COMP:DIMRED’, ‘SAM:DIMRED’, and ‘SDM:DIMRED’ respectively represent the interaction between model complexity and dimensionality reduction, the interaction between sample size and dimensionality reduction, as well as the interaction between the SDM algorithm and dimensionality reduction.


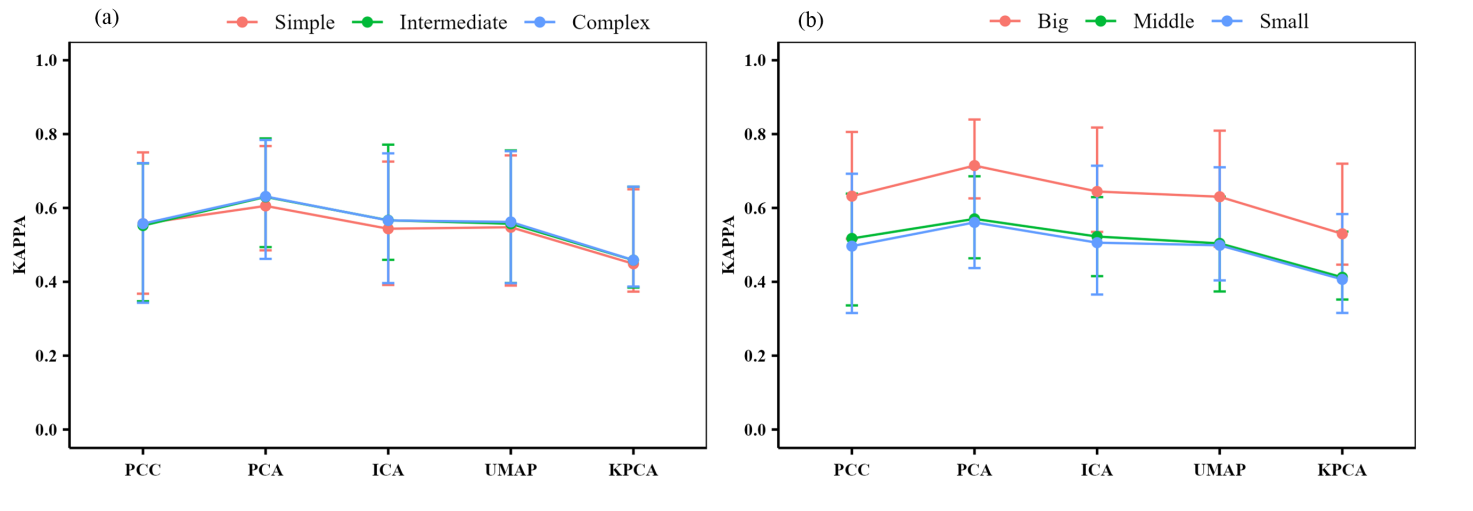


**Figure S9** Statistical results of the predictive performance (measured by KAPPA) of five species distribution models (SDMs, i.e., GLM, GBM, RF, FDA, ANN) under different levels of model complexity (simple, intermediate, and complex) and sample size (small, middle, and big). (a) The median of KAPPA values for all SDMs, based on different model complexity, predicting 32 species distributions under each DRT and PCC. (b) The median of KAPPA values for all SDMs, based on different sample size, predicting 32 species distributions under each DRT and PCC. Based on the sample size (occurrence records) of species, the species are categorized into three groups. Species with less than 100 occurrence records were classified as small, those between 100 and 1,000 records were classified as middle, and those with over 1,000 records were classified as big.


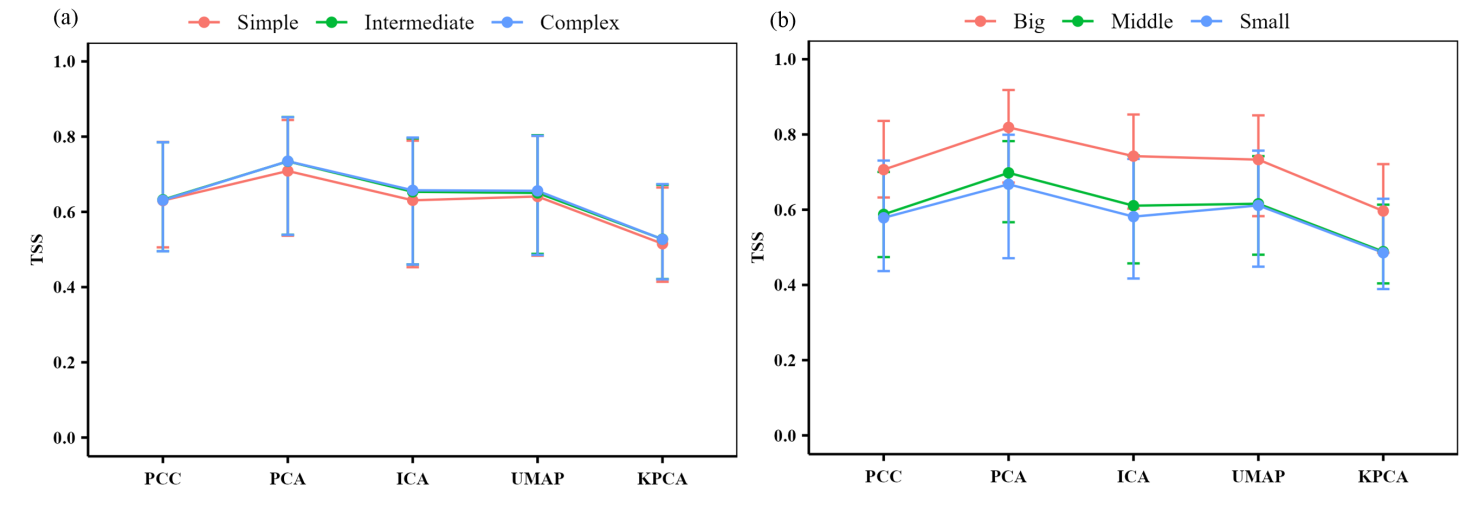


**Figure S10** Statistical results of the predictive performance (measured by TSS) of five species distribution models (SDMs, i.e., GLM, GBM, RF, FDA, ANN) under different levels of model complexity (simple, intermediate, and complex) and sample size (small, middle, and big). (a) The median of TSS values for all SDMs, based on different model complexity, predicting 32 species distributions under each DRT and PCC. (b) The median of TSS values for all SDMs, based on different sample size, predicting 32 species distributions under each DRT and PCC. Based on the sample size (occurrence records) of species, the species are categorized into three groups. Species with less than 100 occurrence records were classified as small, those between 100 and 1,000 records were classified as middle, and those with over 1,000 records were classified as big.

**Parameter selection for the RF and GBM when consider the model complexity**

For RF and GBM, we set up two sets of comparison experiments of important parameters to determine the parameters considered for the final model complexity, respectively. In RF, the minimum number of observations in the terminal nodes (‘nodesize’) was first selected as the parameter for the study of model complexity, and the number of variables randomly sampled as candidates at each split (‘mtry’) was set to a fixed value of 10. Then select ‘mtry’ as the parameter to study model complexity and set the ‘nodesize’ to a fixed value of 20. In GBM, the total number of trees (‘n.trees’) was first selected as the parameter for the study of model complexity, and the maximum depth of each tree (‘interaction.depth’) was set to a fixed value of 2. Then select ‘interaction.depth’ as the parameter to study model complexity and set the ‘n.trees’ to a fixed value of 1000. We finally adopted ‘mtry’ as the parameter in RF and ‘interaction.depth’ as the parameter in GBM when considering the model complexity, according to the comparison of the predictive performance (measured by AUC) of the model under the two sets of parameters (Figure S11). Specifically, we adjusted ‘mtry’ as 15, 10, and 5 to vary the complexity of RF, ‘interaction.depth’ to 1, 2, and 3 for different levels of GBM complexity.

**
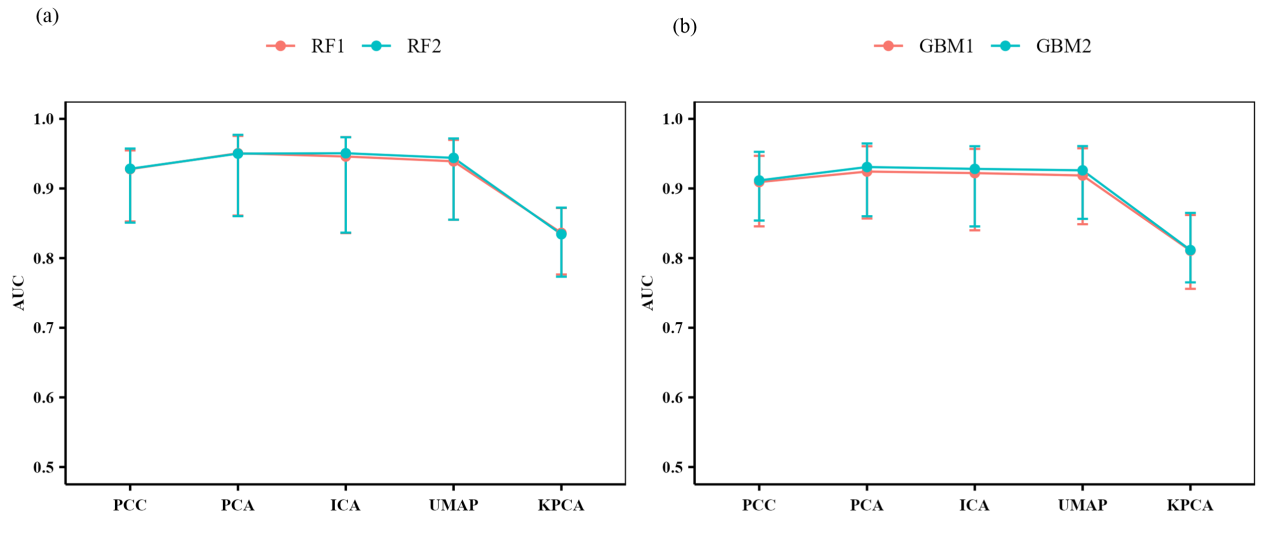
**

**Figure S11** The comparison of the predictive performance (measured by AUC) of the model (RF and GBM) under the two sets of parameters. (a) The median of AUC values for RF1 and RF2, predicting 32 species distributions under each DRT and PCC. (b) The median of AUC values for GBM1 and GBM2, predicting 32 species distributions under each DRT and PCC. RF1 stands for ‘nodesize’ was selected as the parameter for the study of model complexity, and ‘mtry’ was set to a fixed value of 10. RF2 stands for ‘mtry’ was selected as the parameter to study model complexity and set the ‘nodesize’ to a fixed value of 20. GBM1 stands for ‘n.trees’ was selected as the parameter for the study of model complexity, and ‘interaction.depth’ was set to a fixed value of 2. GBM2 stands for ‘interaction.depth’ was selected as as the parameter to study model complexity and set the ‘n.trees’ to a fixed value of 1000.
